# Konjac petroleum ether extract inhibits triple-negative breast cancer cell migration and invasion by attenuating *OLFML2A*-mediated epithelial-mesenchymal transition

**DOI:** 10.3389/fphar.2026.1734640

**Published:** 2026-05-15

**Authors:** Yian Chen, Qinghong Yu, Haining Ding, Wanzhi Jiang, Xiufei Gao

**Affiliations:** The First Affiliated Hospital of Zhejiang Chinese Medical University, Hangzhou, Zhejiang, China

**Keywords:** Amorphophallus konjac, breast cancer, epithelial-mesenchymal transition, OLFML2A, triple-negative breast cancer

## Abstract

**Background and Aim:**

This study, alongside clinical specimens, investigated the inhibitory effect of konjac petroleum ether extract on the migration, invasion, and epithelial-mesenchymal transition (EMT) of triple-negative breast cancer (TNBC) cells via the *OLFML2A* gene, with the objective of elucidating the potential efficacy and mechanism of action of konjac petroleum ether extract.

**Experimental Procedure:**

Histopathological examination of *OLFML2A* was performed on TNBC tumor specimens (n = 8). mRNA and protein levels of *OLFML2A* and EMT markers (*E-cadherin*, *N-cadherin*, *vimentin*, *elastin*) were analyzed via qPCR and Western blot. *OLFML2A*-knockout MDA-MB-231 cells were treated with gradient concentrations of konjac petroleum ether extract. Cell migration (wound healing assay), adhesion, and invasion (Transwell assay) were evaluated, and EMT marker expression was further examined using qPCR, Western blot, and immunofluorescence (n = 3).

**Results:**

In TNBC patients with high *OLFML2A* expression, reduced *E-cadherin* and increased *N-cadherin*, *vimentin*, and *elastin* were observed, while low *OLFML2A* cases exhibited no significant EMT changes. In MDA-MB-231 cells, *OLFML2A* knockdown significantly reduced migration and invasion while upregulating *E-cadherin* and downregulating mesenchymal markers, phenocopying the effects of konjac petroleum ether extract treatment. Konjac extract inhibited cell proliferation with an IC_50_ of 220 μg/mL at 48 h. The extract inhibited migration and invasion in a dose-dependent manner, with significant effects observed at 200 μg/mL, though potential contributions from cytotoxicity at 300 μg/mL cannot be entirely excluded.

**Conclusion:**

The research findings suggest that konjac petroleum ether extract inhibits TNBC cells migration and invasion by modulating *OLFML2A* to inhibit EMT. These findings highlight its potential as a candidate for further investigation for preventing TNBC metastasis, though the contribution of cytotoxicity at higher concentrations warrants further investigation.

## 1 Introduction

Breast cancer is the most prevalent malignancy globally. In recent decades, the incidence of breast cancer has been steadily rising (Montazeri, 2024; Arnold et al., 2022; Lei et al., 2021; Siegel et al., 2024). Cancer cell metastasis is a primary factor that significantly diminishes the survival and prognosis of breast cancer patients (Siegel et al., 2024; Doodmani et al., 2025; Park et al., 2022). TNBC is a cancer subtype characterised by the absence or low expression of oestrogen receptor (ER), progesterone receptor (PR), and human epidermal growth factor receptor (HER2). It constitutes roughly 15%–20% of all breast cancers and is the most aggressive and diverse of all breast cancer subtypes (Obidiro et al., 2023; Polley et al., 2021; Ferrari et al., 2022; Gupta et al., 2020). In addition to chemotherapy and surgery, there is presently an absence of effective therapeutic modalities for TNBC (Obidiro et al., 2023; Maqbool et al., 2022), and patients with this condition frequently exhibit resistance to chemotherapy during the treatment process (Inayatullah et al., 2024; Kumar et al., 2023). Consequently, identifying pertinent metastasis markers in TNBC and investigating the potential molecular pathways are essential for the early management of the disease and the forecasting of its progression (Kumar et al., 2023; Chakraborty and Banerjee, 2023).

EMT is a biological process wherein epithelial cells relinquish their epithelial traits and adopt mesenchymal properties to augment their invasiveness (Fontana et al., 2024; El-Ashmawy et al., 2024). Throughout this process, the expression of the epithelial marker *E-cadherin* diminishes, whilst the expressions of mesenchymal markers, including *N-cadherin* and *Vimentin*, escalate (Wudtiwai et al., 2023). It is frequently associated with tumour invasion, migration, metastasis, and resistance to treatment, serving as a significant precursor to tumour cell metastasis (Fontana et al., 2024; Singh and Siddique, 2024; Long et al., 2025). TNBC is typically associated with the EMT pathway, which confers significant invasive and metastatic properties to TNBC (Wudtiwai et al., 2023; Terragno et al., 2024; Grasset et al., 2022; Burton et al., 2018). The EMT process of TNBC may be obstructed by certain pharmaceuticals and their associated metabolites. Asiaticoside reduces the occurrence of EMT in TNBC via obstructing TGF-β/SMAD signalling (Huang et al., 2023). GSK3β inhibitors reduce the formation of EMT in TNBC via affecting cancer stem cells (CSC) (Vijay et al., 2019).

Traditional Chinese medicine and its active metabolites possess significant promise in impeding the advancement of breast cancer (Zhang et al., 2023a). *Amorphophallus konjac K. Koch ex N.E.Br [Araceae; Amorphophalli rhizoma]* (APR) is a natural traditional Chinese medicinal substance from the *Araceae* family. It has been utilised since antiquity to address different ailments, including elephantiasis, tumours, haemorrhoids, and inflammation (Islam et al., 2023). In traditional Chinese medicine, it was initially documented in the *“Shen Nong Materia Medica”* and it possesses properties that resolve phlegm and detoxify, enhance blood circulation and eliminate blood stasis, as well as block cancerous poisons (Chua et al., 2010). Contemporary pharmacological studies reveal that the primary constituent of konjac is *konjac glucan* (KG) (Kapoor et al., 2024), which has antibacterial and antioxidant properties (Waresindo et al., 2023). It enhances metabolic health by facilitating weight control, stabilising blood glucose levels, and elevating blood lipid profiles (Jain et al., 2025). Research indicates that konjac possesses substantial antioxidant and anti-cancer properties (Raj et al., 2022). Konjac extract can cause cell death and impede the migration of breast cancer cells (Majumder et al., 2021). Our team’s prior research suggested that konjac can impede the growth, proliferation, migration, and invasion of breast cancer via the PI3K/AKT pathway (Li et al., 2022). In TNBC, konjac suppresses the proliferation, migration, invasion, and metastasis of TNBC cells via the PI3K/AKT/mTOR pathway (Wu et al., 2018) and serves as a possible anti-cancer agent.

Olfactomedin-like 2A (*OLFML2A*), often referred to as photomedins-1, is classified within subfamily IV of proteins that possess the olfactory tube protein domain (Ma et al., 2021). Recent research indicates that the overexpression of *OLFML2A* may serve as a prognostic indicator of unfavourable outcomes in acute myeloid leukaemia (AML) (Lu et al., 2023) and is positively associated with the pathological grade of glioma via the Wnt/β-Catenin signalling pathway (Ma et al., 2021). Our team’s prior research indicated that the overexpression of *OLFML2A* may be associated with the proliferation, migration, and invasion of TNBC (Gao X. et al., 2022). Additionally, our previous work demonstrated that konjac extract inhibits TNBC progression through the PI3K/AKT pathway (Li et al., 2022), raising the possibility of crosstalk between this pathway and *OLFML2A*. Furthermore, *OLFML2A* has been identified as a downstream target of activator protein-1 (AP-1) in TNBC (Aubin et al., 2022), a transcription factor known to regulate EMT-related genes. Konjac can induce cell cycle arrest in TNBC cells by downregulating the expression of associated genes such as *OLFML2A*, hence limiting cell proliferation and migration (Wu et al., 2019). These observations suggest that *OLFML2A* may promote EMT through interactions with multiple signaling networks, though direct evidence in TNBC remains limited.

Our team frequently employs konjac in the treatment of patients with TNBC and has observed a notable clinical anti-cancer impact; nevertheless, the precise mechanism remains undefined. Consequently, this study examined the impact of konjac petroleum ether extract on the inhibition of the EMT process and metastasis in TNBC. In conjunction with pertinent clinical specimens, we demonstrated for the first time that the *OLFML2A* gene influences the invasion, migration, and metastasis of TNBC via the EMT pathway. The petroleum ether extract of konjac suppresses EMT and metastasis in TNBC cells via diminishing the activity of *OLFML2A*. Our research findings suggest that konjac may hold promise as a therapeutic agent for patients with TNBC though further investigation is needed.

Based on the above findings and the existing gaps in knowledge, the present study was designed with two primary objectives. The first and main objective was to elucidate the role of *OLFML2A* in regulating EMT, migration, and invasion in TNBC. By analyzing clinical tissue specimens and employing gain- and loss-of-function approaches in TNBC cell lines, we aimed to determine whether *OLFML2A* expression correlates with EMT marker alterations and functional changes in tumor cell behavior. The secondary objective was to evaluate the therapeutic potential of RhA in modulating TNBC progression. Specifically, we sought to investigate whether RhA exerts its inhibitory effects on TNBC cell proliferation, EMT, migration, and invasion through the modulation of *OLFML2A*. By integrating clinical observations with *in vitro* mechanistic studies, this work seeks to provide a comprehensive understanding of the *OLFML2A*-mediated EMT axis and its targeting by a natural botanical extract, thereby offering a potential therapeutic strategy for TNBC intervention.

## 2 Materials and methods

### 2.1 Preparation of konjac petroleum ether extract

The traditional Chinese medicine utilised, Konjac (Batch number: 2004192), originates from Zhejiang, China. Pulverise the medicine Konjac into a fine powder and reserve it. The plant specimens were identified by Dr. Zhu Bo, a Chinese medicine expert, and is stored in the specimen room of the First Affiliated Hospital of Zhejiang Chinese Medicine University with the voucher number: MY166. To comply with the ConPhyMP checklist (Heinrich et al., 2022), multiple chemical profile characterization methods should be employed. In this study, the Konjac extract from the same source has been previously characterized through Gas Chromatography-Mass Spectrometry (GC-MS), Liquid Chromatograph Mass Spectrometer (LC-MS) and Thin-Layer Chromatography (TLC). Furthermore, we conducted a comprehensive analysis of the extract’s properties to ensure that it could produce a reproducible characteristic spectrum. These analyses identified a variety of bioactive compounds, including Undecylenic acid and Eicosadienoic acid. Weigh 500 g of Konjac powder, add 3 L of 95% ethanol, heat and reflux for extraction for 1 h, filter using filter paper, collect the filtrate, and repeat this procedure a total of three times. The three filtrates were amalgamated in a 2 L round-bottom flask, concentrated under reduced pressure at 45 °C to approximately 300 mL, and subsequently transferred to a 500 mL round-bottom flask. The residue was washed with 95% ethanol and thereafter transferred in multiple sections to a 500 mL round-bottom flask. The substance was concentrated under decreased pressure at 65 °C and subsequently dried to get the alcohol extract F1 of hexachlorosilum. The crude extract was acquired by weighing 10.8246 g.

Weigh 8 g of the F1 sample, suspend it in about 500 mL of distilled water, transfer the mixture to a separating funnel, add an equivalent volume of petroleum ether, secure the stopper to ensure thorough mixing, then gradually release the lower aqueous phase and decant the petroleum ether layer from the upper orifice. Reintroduce the aqueous phase into the separating funnel and do two extractions with an equivalent volume of petroleum ether. Consolidate the three petroleum ether extracts. The fraction of petroleum ether acquired during vacuum evaporation at 30 °C is designated as F2. The petroleum ether layer was initially concentrated under reduced pressure at 35 °C, subsequently transferred to a 10 mL round-bottom flask (3.423 g), and dried under reduced pressure at 60 °C. It was measured at 9.12 g and preserved in a refrigerator at 4 °C for subsequent utilisation. corresponding to a DER of 146:1.

### 2.2 Tissue specimens

We obtained 18 tissue specimens from patients with TNBC who received surgical therapy at Zhejiang Provincial Hospital of Traditional Chinese Medicine between 2019 and 2024. All breast cancer patients did not have preoperative chemotherapy or radiotherapy. All tissue specimens were histologically validated by pathologists.

### 2.3 Immunohistochemistry

Section tissues to a thickness of five microns, thereafter disperse them in water at 42 °C, and then incubate in an oven at 60 °C for 30 min. Dewaxing was conducted in xylene, rehydration was executed in graded alcohols, and subsequent washing was performed with distilled water five times. After 5–8 min of microwave thermal treatment with EDTA, allow it cool to room temperature. Administer the blocking serum and incubate at 37 °C for 30 min, then dispose of the surplus serum. Incorporate anti-*OLFML2A* (NBP1-56749, 1:1000, Novus, United States), *E-cadherin* (ab231303, 1:500, Abcam, United Kingdom), and *N-cadherin* (ab280375, 1:500, Abcam) The principal antibodies from Britain, *Vimentin* (ab92547, 1:500, Abcam, Britain) and *Elastin* (ab213720, 1:500, Abcam, Britain), were incubated overnight at 4 °C. Utilise a microscopic camera to capture photographs. The staining intensity was assessed via ImageJ software.

Immunohistochemical analysis was performed on tissue sections from 18 TNBC patients (biological replicates). For each patient, three non-consecutive sections were analyzed (technical replicates), and five random fields per section were quantified.

### 2.4 Immunofluorescence

The cells were fixed with 4% paraformaldehyde for 10 min, followed by three rinses of the slides with ice-cold PBS. After incubating with 0.1% Triton X-100 for 10 min, rinse thoroughly with PBS. The sample was obstructed using a 1% BSA and glycine solution, followed by the addition of anti-*OLFML2A* (NBP1-56749, 1:4000, Novus, United States), *E-cadherin* (ab231303, 1:4000, Abcam, United Kingdom), and *N-cadherin* (ab280375). The primary antibodies, *Vimentin* (ab92547, 1:4000) and *Elastin* (ab213720, 1:4000), both from Abcam, Britain, were incubated overnight at 4 °C. Subsequently, after three washes with PBST, incubate the secondary antibody in darkness. Conclude by staining with DAPI (1 μg/mL) for 1 min, followed by three washes with PBS, sealing with an anti-quenching mounting medium, and capturing images with a microcamera.

### 2.5 Cell creep

The cells in the culture dish were digested and resuspended in 0.25% EDTA, and the digestion was halted with DMEM high glucose complete medium. Position polylysine-coated climbing plates within 6-well plates, thoroughly resuspend and distribute the cells into the plates, introduce DMEM high glucose complete media, and conduct standard cell culture in a 37 °C incubator with 5% CO2. Following a 24-h period, the fusion rate of the cell crawling slides was recorded at 90%, after which the slides were extracted.

### 2.6 Cell culture and grouping

The human TNBC cell line (MDA-MB-231) was acquired from Yuan Jing Corporation (China) and maintained in DMEM media supplemented with 10% foetal bovine serum and additional additives. The cells were grown in an incubator at 37 °C with 5% CO2. Upon achieving 80%–90% cell fusion, the cells were subcultured at a 1:2 ratio, subjected to digestion with 0.25% trypsin for 1 min, centrifuged, collected, resuspended, and inoculated.

MDA-MB-231 cells were inoculated into a six-well plate at a density of 8 × 10^5 per well, incubate for 24 h, then discard the culture media. Different incubation times were selected based on specific experimental objectives. For wound healing and Transwell invasion assays, a 24-h incubation was chosen for the following reasons: (i) Migration kinetics: MDA-MB-231 cells close wounds within 24–36 h under control conditions; longer incubation would eliminate the ability to detect differences between groups. (ii) Proliferation confounding: Prolonged incubation increases the contribution of cell proliferation to wound closure, complicating interpretation of true migration effects. The 24 h time point minimizes this confounding factor. (iii) Matrigel integrity: In Transwell assays, Matrigel coating begins to degrade after 24–30 h, potentially allowing passive cell passage and compromising invasion specificity. These time points are consistent with established standards in TNBC migration and invasion research (Chien et al., 2025; Nguyen et al., 2023; Wang et al., 2024). All wound healing assays were performed with three independent biological replicates (n = 3), each with technical triplicates (3 wells per condition, with three to five images per well).

For treatment experiments, MDA-MB-231 cells were divided into the following groups: MDA-MB-231: Cells treated with medium containing 0.1% DMSO (vehicle control). MDA-MB-231_KO (KO): MDA-MB-231 cells were transduced with lentiviral particles containing CRISPR/Cas9 constructs targeting the *OLFML2A* gene. After puromycin selection, single-cell clones were expanded and validated by Western blot and qPCR. MDA-MB-231_OE (OE): MDA-MB-231 cells were transfected with an *OLFML2A* overexpression plasmid using Lipofectamine 3000. Stably transfected cells were selected with G418 and validated by Western blot and qPCR. Ctrl OE: For comparison, wild-type MDA-MB-231 cells (untransfected) and cells transfected with empty vector (EV) or non-targeting siRNA (si-NC) were used as appropriate controls. 100 μg/mL: Cells treated with RhA at a final concentration of 100 μg/mL 200 μg/mL: Cells treated with RhA at a final concentration of 200 μg/mL 300 μg/mL: Cells treated with RhA at a final concentration of 300 μg/mL. All treatments were applied for 24 h (for migration, invasion, and EMT marker analysis) or 48 h (for cytotoxicity assays), as specified in the respective experimental protocols. For treatment experiments, RhA was dissolved in DMSO and diluted in culture medium to achieve final concentrations of 100, 200, and 300 μg/mL. The final DMSO concentration in all treatment groups was 0.1%. Control cells were treated with culture medium containing 0.1% DMSO alone (vehicle control).

All *in vitro* experiments were performed with three independent biological replicates, each conducted on different days using different cell passages. For each biological replicate, technical triplicates were included for all quantitative assays as specified below.

### 2.7 Cell transfection

One day before to virus transfection, cells in the logarithmic growth phase were dissociated into a single-cell suspension and subsequently seeded into 6-well plates, with a culture medium volume of 2 mL per well. The cells were incubated at 37 °C with 5% CO2 for 24 h to attain a confluence of 30%–50% before to transduction. Remove the virus from −80 °C and store it on ice until fully thawed. Introduce a suitable quantity of the virus into the cell culture media, then including Polybrene to enhance viral infection. Subsequent to 24 h of viral transduction, substitute with new complete medium.

For *OLFML2A* overexpression, MDA-MB-231 cells were transfected with an *OLFML2A* overexpression plasmid using Lipofectamine 3000 according to the manufacturer’s instructions. Cells transfected with the empty vector (pcDNA3.1) served as the control group. For siRNA-mediated knockdown, cells were transfected with *OLFML2A*-targeting siRNA (si-*OLFML2A*) or negative control siRNA (si-NC) using Lipofectamine RNAiMAX. For CRISPR/Cas9-mediated knockout, cells were transduced with lentiviral particles containing *OLFML2A*-targeting guide RNAs. Wild-type (untransfected) MDA-MB-231 cells were used as controls for knockout experiments.

The utilised SiRNA sequence is as follows: 5′-TCA​CCT​ACA​CCC​TCC​ACT​TC-3′ (*Olfml2a*-SiRNA). The empty vector plasmid, *OLFML2A* overexpression plasmid, *OLFML2A* small interfering RNA (si-*OLFML2A*), and small interfering RNA negative control (si-NC) utilised in this investigation were all developed and synthesised by Guangzhou Yuanjing Biology Company.

### 2.8 Cytotoxicity determination

Count the cells and inoculate them into 96-well plates, with 3,000 cells per well. The cells were cultured at 37 °C and 5% CO2 for 24 h. Discard the original culture medium and add 200 μL of culture medium containing varying concentrations of Serpentine petroleum ether extract (100, 150, 200, 250, 300, 350, 400, 450, 500, 550, 600 μg/mL), then incubate for an additional 24 h. Subsequently, add 20 μL of CCK-8 reagent to each well and incubate. Cell viability was assessed by measuring absorbance at 450 nm using a microplate reader at 0.5, 1, 1.5, and 2 h. The cell-free group of CCK-8 solution served as the blank group, while the untreated cell group of CCK-8 solution functioned as the control group for calculating cell viability.

For each biological replicate (n = 3), six technical replicate wells were used per concentration. Cell viability was calculated as the mean of the six technical replicates, and the mean of three biological replicates was used for statistical analysis.

### 2.9 Real-time quantitative PCR analysis

Total RNA was extracted from cells and tissues using the Steady Pure rapid RNA Extraction Kit (AG21023, AG, China). Reverse transcription of the extracted RNA was conducted with the Evo M-MLV reverse transcription premixed kit, which includes a gDNA removal reagent (AG11728, AG, China), and the SYBR® Green Pro Taq HS premixed qPCR kit (AG11701, AG) was utilised for qPCR testing in China. The relative mRNA expression in each sample was determined using the 2^−ΔΔCT^ method, and results were normalised by calculating the fold changes based on the RNA level of *GAPDH* in the same sample. Table 1 presents the upstream and downstream primers for qPCR synthesis.

**TABLE 1:** Primer information.

Name of primer	Sequences (5′–3′)
E-cadherin-F	GAG​TGC​CAA​CTG​GAC​CAT​TCA​GTA
E-cadherin-R	AGT​CAC​CCA​CCT​CTA​AGG​CCA​TC
N-cadherin-F	AGC​ACA​GTG​GCC​ACC​TAC​AAA​G
N-cadherin-R	CAG​CTC​CTG​GCC​CAG​TTA​CA
Vimentin-F	AAG​ACG​GTT​GAA​ACT​AGA​GAT​GGA​C
Vimentin-R	TGC​TGG​TAA​TAT​ATT​GCT​GCA​CTG​A
Elastin-F	TCC​AGG​TGT​AGG​TGG​AGC​TT
Elastin-R	GTA​GGG​CAG​TCC​ATA​GCC​AC
Olfml2a-F	AGC​CTG​GAT​GAA​GGA​CCC​TG
Olfml2a-R	GAA​GGC​GCG​GTT​GTA​GTA​GA
GAPDH-F	GCA​CCG​TCA​AGG​CTG​AGA​AC
GAPDH-R	TGG​TGA​AGA​CGC​CAG​TGG​A

For patient samples, qPCR was performed in duplicate technical reactions for each of the 18 biological replicates. For cell line experiments, three independent biological replicates were performed, each with triplicate technical reactions.

### 2.10 Western blot

Introduce the prepared lysis buffer (RIPA lysis buffer: protease inhibitor: phosphatase inhibitor = 100:1:1) to the cell or tissue precipitate, mix completely, incubate on ice for 20 min, then centrifuge at 4 °C and 12,000 RPM for 10 min, and collect the supernatant. The protein content was assessed by the BCA protein quantification kit (AR0146, BOSTER, China), the samples were resolved via 10% SDS-PAGE gel (PG112, Yamei, China), and subsequently transferred to the PVDF membrane (FFP28, Beyotime, China) upon completion of the mould transfer. Following a 20-min incubation with the fast blocking solution (P0252, Beyotime, China), the membrane was subsequently incubated with *OLFML2A* (ab75882, 1:1000, Abcam, Britain) and *E-cadherin* (ab231303, 1:1000, Abcam). *N-cadherin* (ab280375, 1:1000, Abcam, Britain), *Vimentin* (ab92547, 1:1000, Abcam, Britain), *Elastin* (ab213720, 1:1000, Abcam, Britain), and GAPDH (sc47724, 1:1000, Santa Cruz, United States) were incubated overnight at 4 °C. Upon completion of incubation, rinse the membrane thrice with TBST, allowing each wash to last 10 min. Incubate with the suitable horseradish peroxidase (HRP) conjugated secondary antibody (7074P2, 7076P2, 1:400, CST, United States) at ambient temperature for 60 min, followed by three washes of the membrane with TBST, each lasting 10 min. The mixed ECL luminescent solution (FD8000, FDbio, China) was introduced, and images were acquired utilising a chemiluminescence imaging equipment (BIO-RAD, United States). The stripe density was assessed via ImageJ software (version 4.2). For quantification, the integrated density of each protein band was measured using ImageJ software (version 4.2) after background subtraction. The expression level of *OLFML2A* was normalized to that of GAPDH from the same sample lane. Normalized values were then used for statistical analysis.

For patient samples, Western blot was performed on lysates from each of the 18 individual patients (biological replicates). For cell line experiments, three independent biological replicates were performed, each representing a separately cultured and treated cell population.

### 2.11 Statistical analysis

All statistical analyses were performed using GraphPad Prism software (version 9.5.1, GraphPad Software, San Diego, CA, United States). Data are presented as mean ± standard deviation (SD) unless otherwise specified. The normality of data distribution was assessed using the Shapiro–Wilk test. For datasets that did not pass the normality test or had small sample sizes (n < 10 per group), non-parametric tests were applied.

For comparisons between two independent groups, the Mann–Whitney U test was used. For paired samples (e.g., cancer tissue vs. adjacent tissue from the same patient), the Wilcoxon signed-rank test was applied. For comparisons involving more than two groups, the Kruskal–Wallis test was performed, followed by Dunn’s *post hoc* test for multiple comparisons when the overall test indicated significant differences (p < 0.05). Dunn’s test corrects for multiple comparisons using the Bonferroni method. For correlation analyses, Spearman’s rank correlation coefficient was computed to assess the strength and direction of associations between variables, as this method makes no assumptions about linearity or normal distribution.

All statistical tests were two-tailed, and a p-value of less than 0.05 was considered statistically significant. In all figures, significance levels are indicated as follows: ***p < 0.001, compared with control group; **p < 0.01, compared with control group; *p < 0.05, compared with control group; ###*p* > 0.05, compared with control group. All statistical tests and sample sizes are specified in the corresponding figure legends.

### 2.12 Data source

Data from two major databases, the Human Protein Atlas (HPA) and The Cancer Genome Atlas (TCGA), were employed to investigate the significance of *OLFML2A* in breast cancer. The HPA offers extensive data on the expression patterns of proteins across diverse human tissues at both the protein and mRNA levels. It provides high-quality immunohistochemistry data, facilitating the visualisation of protein localisation in both normal and cancerous tissues. This study utilised HPA data to examine the normal tissue expression profile of *OLFML2A*, providing a reference for elucidating its expression alterations in breast cancer. The TCGA database has extensive multi-omics data, including gene expression profiles from many cancer patients. We obtained gene expression data (quantified as normalised transcripts per million, nTPM) from TCGA for breast cancer research. Breast cancer specimens were acquired from the TCGA - BC cohort. The data were utilised to examine the expression level of *OLFML2A* in breast cancer tissues and its association with other genes and immune cell infiltration.

### 2.13 Data analysis

We concentrated on the correlation study between *OLFML2A* and various EMT genes, including *N-cadherin*, *vimentin* and *elastin*. The Spearman rank correlation coefficient was computed to assess the strength and direction of the link between the expression levels of these genes.

We employed the ssGSEA (single-sample gene-set enrichment analysis) program to examine the link between *OLFML2A* expression and immune cell infiltration. This approach enabled us to assess the relative number of several immune cell types in each breast cancer sample utilising gene expression data. Subsequently, Spearman’s correlation analysis was used to investigate the association between the estimated levels of immune cell infiltration and *OLFML2A* expression. The resultant correlation coefficients (R) and p-values were employed to evaluate the significance of these correlations. A positive R value signifies a positive association, indicating that an increase in *OLFML2A* expression corresponds with a rise in the infiltration level of the relevant immune cell type, whereas a negative R value denotes the opposite relationship.

All statistical analyses were performed utilising R software, incorporating pertinent programs such as “corrplot” for correlation visualisation and “GSVA” for ssGSEA analysis.

## 3 Results

### 3.1 *OLFML2A* is significantly upregulated in the cancerous tissues of individuals diagnosed with TNBC

**FIGURE 1:**
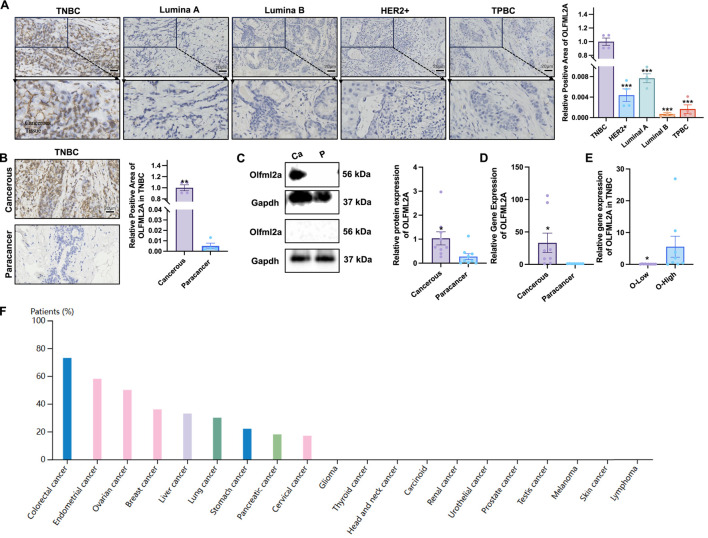
*OLFML2A* is significantly upregulated in the cancerous tissues of individuals diagnosed with TNBC. **(A)** Immunohistochemical analysis of cancer tissues and the corresponding positive rate of *OLFML2A* in patients with various breast cancer types; **(B)** Immunohistochemical analysis of cancer and adjacent tissues in patients with TNBC and the corresponding positive rate of *OLFML2A*; **(C)** Representative Western blot images showing *OLFML2A* protein expression in cancerous and adjacent non-cancerous (Paracancer) tissues from two TNBC patients: one with high *OLFML2A* expression (Patient A, upper panel) and one with low *OLFML2A* expression (Patient B, below panel). GAPDH was used as a loading control. These images illustrate the heterogeneity of *OLFML2A* expression across TNBC patients; **(D)** Variations in mRNA levels of *OLFML2A* in cancer and adjacent tissues of TNBC patients; **(E)** Variations in mRNA levels of *OLFML2A* between eight patients exhibiting high *OLFML2A* expression and 10 patients with low expression; **(F)** The expression of *OLFML2A* in diverse cancer tissues was evaluated using the public database HPA. (*p < 0.05, compared with Paracancer, n = 8 per group).

We assessed the expression of *OLFML2A* in various cancer tissues by analysing data from the public database HPA, revealing that *OLFML2A* expression was elevated in breast cancer (Figure 1F). We collected cancer tissues from clinical breast cancer patients with various subtypes (TNBC, Luminal A, Luminal B, HER2+, TPBC) for immunohistochemistry investigation to assess the differential expression of *OLFML2A*. The findings indicated that the expression of *OLFML2A* in the cancer tissues of TNBC patients was substantially elevated compared to various other breast cancer types (Figure 1A). We additionally collected cancerous and surrounding tissues from TNBC patients for immunohistochemistry examination. The findings indicated that the expression of *OLFML2A* was elevated in the cancerous tissues compared to the surrounding tissues (Figure 1B). To examine the expression disparities of *OLFML2A* in TNBC patients, we assessed the mRNA and protein levels in cancerous and surrounding tissues from 18 clinically obtained TNBC patients. The findings indicated that the expression of *OLFML2A* in cancerous tissues was higher than surrounding tissues in eight patients (Figures 1C,D). The expression of *OLFML2A* in the cancer tissues of the other 10 patients did not exhibit a significant increase (Supplementary Material). Subsequently, the mRNA of *OLFML2A* was identified in 10 patients exhibiting both high and low expression levels of *OLFML2A*. The findings indicated variations in the expression of *OLFML2A* across TNBC patients (*p* < 0.05) (Figure 1E). The results suggest that *OLFML2A* is significantly expressed in the cancerous tissues of patients with TNBC, with variations in expression observed among different specimens.

### 3.2 *OLFML2A* influences the expression of EMT markers in cancerous tissues of individuals with TNBC

**FIGURE 2:**
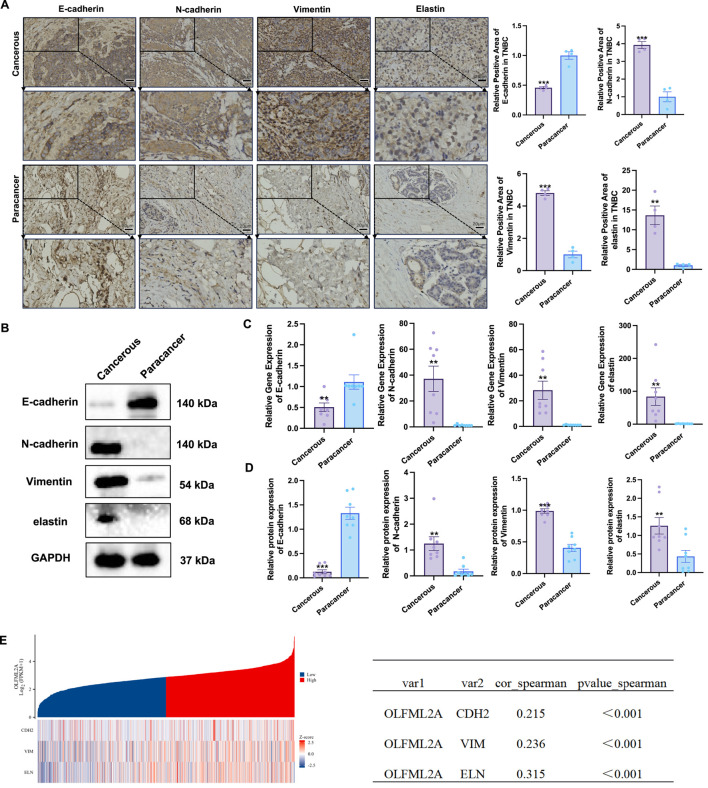
*OLFML2A* influences the expression of EMT markers in cancerous tissues of individuals with TNBC. **(A)** Immunohistochemical analysis of cancerous and adjacent tissues in TNBC patients exhibiting elevated *OLFML2A* expression, along with their corresponding positive rates; **(B)** Variations in protein levels of EMT markers between cancerous and adjacent tissues in TNBC patients with high *OLFML2A* expression (n = 8); **(C)** Differences in mRNA levels of EMT-related markers (n = 8); **(D)** The relative positive rates of EMT-related markers; **(E)** Calculation of the Spearman rank correlation coefficient to assess the relationship between *OLFML2A* and EMT-related genes. (***p < 0.001, compared with Cancerous; **p < 0.01, compared with Cancerous, n = 8 per group).

To investigate the impact of *OLFML2A* on EMT, we conducted immunohistochemistry section analysis on cancerous and surrounding tissues from TNBC patients exhibiting elevated *OLFML2A* expression. To assess the expression variations of EMT-related markers *E-cadherin*, *N-cadherin*, *vimentin*, and *elastin* (Figure 2A). The findings suggest that the expression of the epithelial marker *E-cadherin* in cancerous tissues was notably lower compared to adjacent tissues, while the expressions of the mesenchymal markers *N-cadherin*, *vimentin*, and *elastin* in cancerous tissues were significantly elevated relative to adjacent tissues. Subsequently, we measured the mRNA and protein expression levels of EMT markers in individuals exhibiting elevated *OLFML2A* expression. The test results aligned with immunohistochemistry findings. The expression of the epithelial marker *E-cadherin* in cancerous tissues was reduced compared to neighbor tissues. The expressions of mesenchymal markers *N-cadherin*, *vimentin*, and *elastin* were increased as shown by Western blot analysis in cancerous tissues compared to surrounding tissues (Figures 2B–D). No notable variations in the expression of EMT-related markers were seen between the 10 TNBC cancer tissues with low *OLFML2A* expression and the surrounding tissues (Supplementary Material). We assessed the association between *OLFML2A* and EMT-related genes via gene-gene correlation analysis and computed the Spearman rank correlation coefficient. The findings indicated that *OLFML2A* expression showed moderate positive correlations with the mesenchymal markers *N-cadherin*, p < 0.001, *vimentin*, p < 0.001, and *elastin*, p < 0.001 (Figure 2E). Although these correlations are statistically significant, they cannot fully suggest the difference in the expression of *OLFML2A* and the expression of EMT markers. This indicates that, apart from *OLFML2A*, there are other factors involved in the regulation of EMT in TNBC.

### 3.3 The petroleum ether extract from konjac suppresses the proliferation of TNBC cell lines in a dose-dependent fashion

**FIGURE 3:**
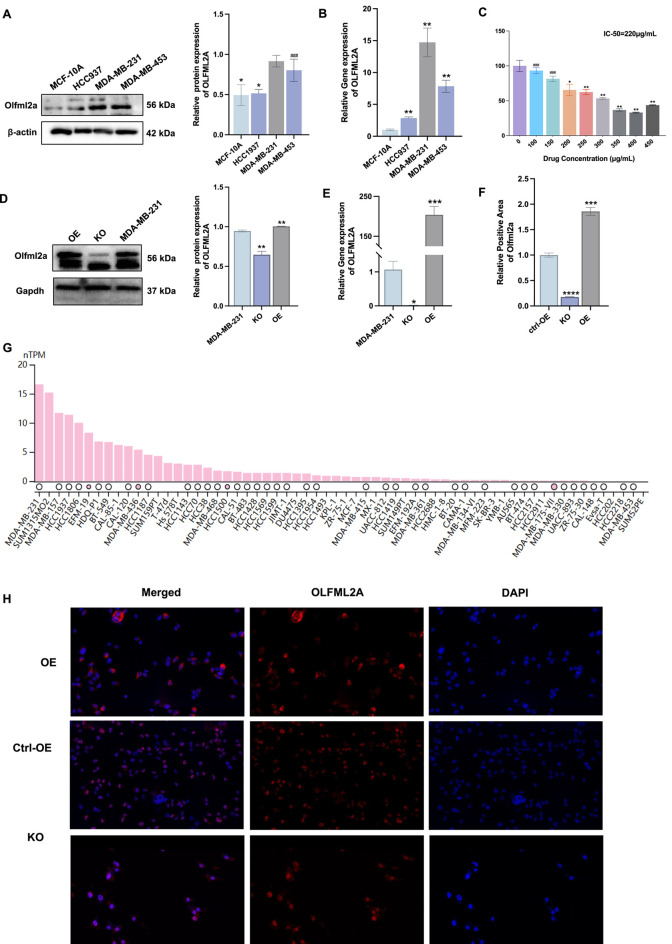
The petroleum ether extract from Konjac suppresses the proliferation of TNBC cell lines in a dose-dependent fashion. **(A)** Protein concentrations of *OLFML2A* across various TNBC cell lines (*p < 0.05, compared with MCF-10A; ###*p* > 0.05, compared with MCF-10A, n = 3); **(B)** mRNA expression variations of *OLFML2A* (**p < 0.01, compared with MCF-10A, n = 3); **(C)** CCK8 assay determines the IC50 of pharmacological effects (**p < 0.01, compared with 0 μg/mL; *p < 0.05, compared with 0 μg/mL; ###*p* > 0.05, compared with 0 μg/mL, n = 3); **(D)** Protein levels of *OLFML2A* following knockout and overexpression in MDA-MB-231 (**p < 0.01, compared with MDA-MB-231, n = 3); **(E)** mRNA expression discrepancies of *OLFML2A* in MDA-MB-231, MDA-MB-231_OE, and MDA-MB-231_KO cell lines (*p < 0.05, compared with MDA-MB-231; ***p < 0.001, compared with MDA-MB-231, n = 3); **(F)** Relative fluorescence intensity of *OLFML2A* (****p < 0.0001, compared with ctrl-OE; ***p < 0.001, compared with ctrl-OE, n = 3); **(G)** Assessment of *OLFML2A* expression levels in diverse cell lines utilising the TCGA database; **(H)** Immunofluorescence employed to evaluate *OLFML2A* expression in cells.

To evaluate the cell line exhibiting elevated *OLFML2A* expression, we assessed the proteins and mRNAs of normal human breast epithelial cells (MCF-10A) and TNBC cells (HCC937, MDA-MB-231, MDA-MB-453). The findings suggested that the expression of *OLFML2A* was notably elevated in the MDA-MB-231 cell line (Figures 3A,B). The study of the TCGA database revealed that the expression of *OLFML2A* in MDA-MB-231 cells exceeded that in other cell lines (Figure 3G). Subsequently, the MDA-MB-231 cell line with *OLFML2A* gene deletion and overexpression was established using lentiviral transfection, and the mRNA and protein of the cell line were validated. The findings demonstrated that the *OLFML2A* gene was effectively overexpressed in MDA-MB-231_OE. The knockout was successfully achieved in MDA-MB-231_KO (*p <* 0.05) (Figures 3D,E). Cellular immunofluorescence studies demonstrated that *OLFML2A* expression was elevated in MDA-MB-231_OE and diminished in MDA-MB-231_KO (Figures 3F,H). Subsequently, we inoculated the cells onto 96-well plates and subjected them to varying doses of Konjac petroleum ether extract for 48 h for the specified duration. The findings indicated that Konjac petroleum ether extract suppressed the proliferation of MDA-MB-231 cells in a dose-dependent fashion, with the concentration may inhibiting 50% of cell growth (IC_50_) value of 220 μg/mL after 48 h of treatment (Figure 3C). The aforementioned results demonstrate that the petroleum ether extract of Konjac may inhibits the proliferation of TNBC cell lines in a dose-dependent fashion.

### 3.4 *OLFML2A* expression influences the EMT in the MDA-MB-231 cell line

**FIGURE 4:**
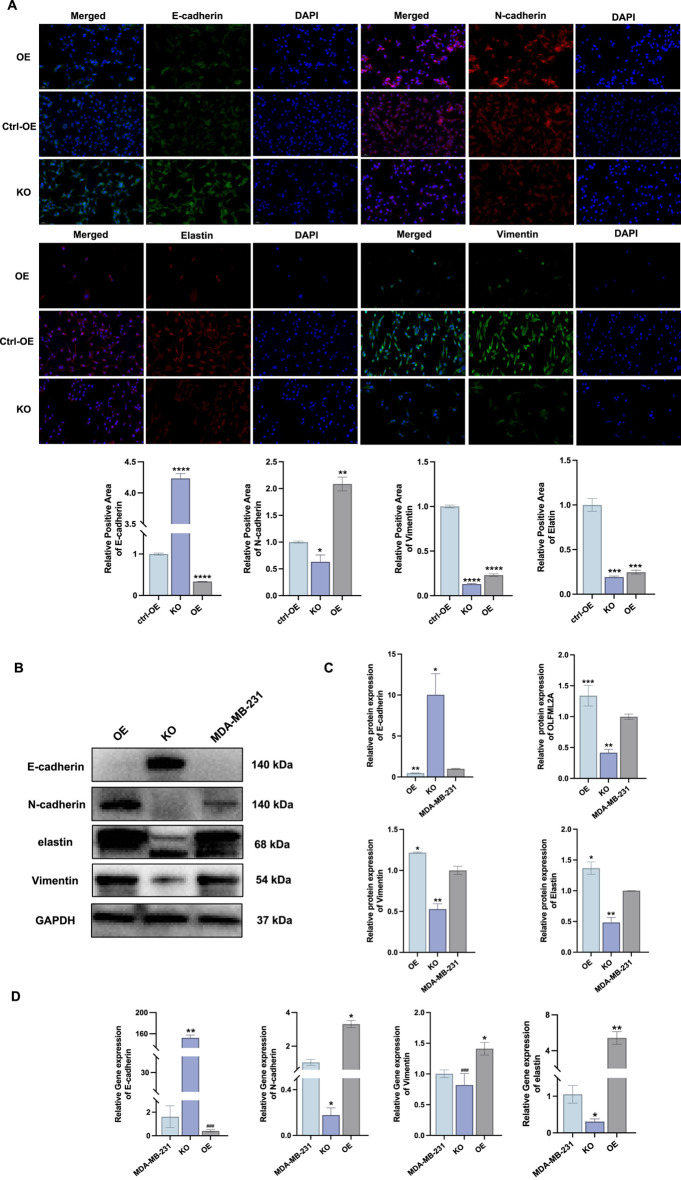
*OLFML2A* expression influences the EMT in the MDA-MB-231 cell line. **(A)** Immunofluorescence was employed to ascertain the expression of EMT markers in the MDA-MB-231, MDA-MB-231_OE, and MDA-MB-231_KO cell lines (****p < 0.0001, compared with ctrl-OE; ***p < 0.001, compared with ctrl-OE; **p < 0.01, compared with ctrl-OE; *p < 0.05, compared with ctrl-OE, n = 3); **(B)** Western blotting was utilised for the detection of EMT markers; **(C)** The relative positivity rate of EMT indicators; **(D)** Variations in mRNA expression of EMT markers. (***p < 0.001, compared with MDA-MB-231; **p < 0.01, compared with MDA-MB-231; *p < 0.05, compared with MDA-MB-231; ###*p* > 0.05, compared with MDA-MB-231, n = 3).

Preliminary studies by our team on *OLFML2A* indicated its potential association with the proliferation, invasion, and migration of TNBC. To elucidate the impact of *OLFML2A* on EMT, we subsequently performed polymerase chain reaction (PCR) and Western blot analysis. The results demonstrated that, in comparison to the negative control group, silencing *OLFML2A* led to an increase in the expression of the epithelial marker *E-cadherin* in the MDA-MB-231 cell line. Nonetheless, the expressions of the mesenchymal markers *N-cadherin*, *vimentin*, and *elastin* diminished. In the cell lines exhibiting *OLFML2A* overexpression, the expression of the epithelial marker *E-cadherin* diminished, whilst the expressions of the mesenchymal markers *N-cadherin*, *vimentin*, and *elastin* escalated (Figures 4B–D). The immunofluorescence detection of MDA-MB-231_KO, MDA-MB-231_OE, and MDA-MB-231 corroborated this conclusion (Figure 4A). The aforementioned finding suggests that the overexpression of *OLFML2A* may facilitate EMT in TNBC, whereas the suppression of *OLFML2A* may impede EMT in TNBC. *OLFML2A* may serve as a possible target for the EMT process in TNBC (Knockout cells were analyzed at 48 h post-validation, while treated cells were analyzed at 24 h post-treatment).

### 3.5 RhA and the silencing of *OLFML2A* impede the invasion and migration of MDA-MB-231 cells

**FIGURE 5:**
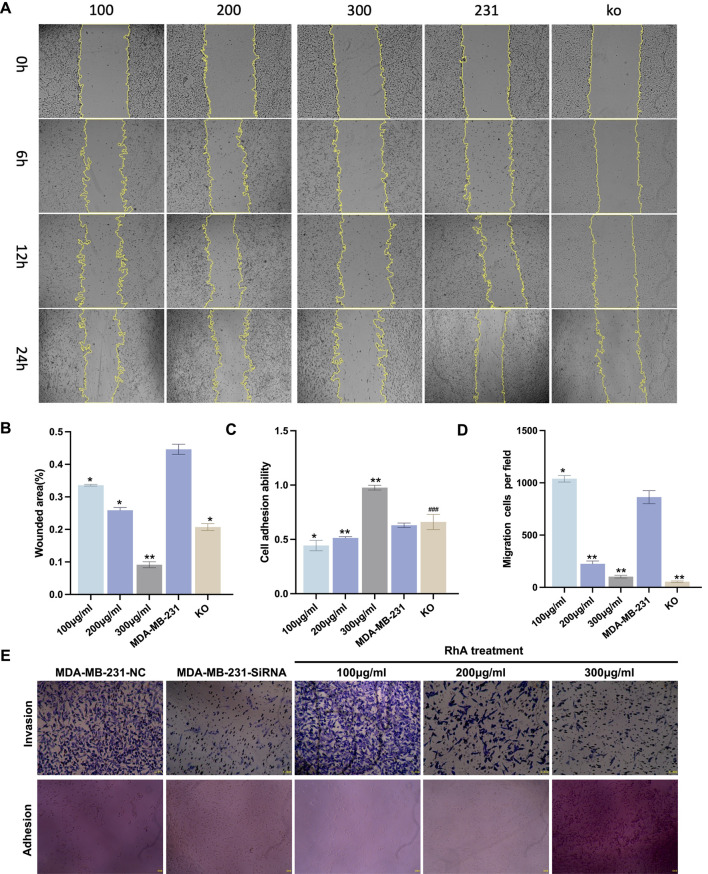
The petroleum ether extract of Konjac (RhA) and the silencing of *OLFML2A* impede the invasion and migration of MDA-MB-231 cells. **(A)** The wound healing experiment was performed to examine wound closure at 0, 6, 12, and 24 h; **(B)** wound healing rate. **(C)** Cell adhesion rate **(D)** Cell invasion area **(E)** The Transwell cell invasion test and adhesion assay were employed to examine the impact of *OLFML2A* and konjac petroleum ether extract on the invasion and migratory capabilities of cells. (**p < 0.01, compared with MDA-MB-231; *p < 0.05, compared with MDA-MB-231; ###*p* > 0.05, compared with MDA-MB-231. All wound healing assays were performed with three independent biological replicates (n = 3), each with technical triplicates (3 wells per condition, with three to five images per well). Data are presented as mean ± SD from three independent experiments (n = 3). Statistical analysis was performed using Kruskal–Wallis test followed by Dunn’s multiple comparison test for multi-group comparisons, and Mann-Whitney U test for two-group comparisons. *p < 0.05, **p < 0.01, **p < 0.001 compared to control, n = 3).

*OLFML2A* is significantly expressed in MDA-MB-231 cells, potentially correlating with the enhanced invasion and migratory capabilities of TNBC. Consequently, we utilised the MDA-MB-231 cell line as a model and contrasted it with the RhA administration group and the *OLFML2A* gene knockout group to further investigate the effects of the *OLFML2A* gene and RhA on the invasion and migration of TNBC cell lines. The wound healing assay was employed to assess the migratory capacity of MDA-MB-231 cells under various settings. The state of wound closure was assessed at 0, 6, 12, and 24 h, respectively. The results indicated that RhA impeded wound closure, with the wound healing rate progressively declining as concentration increased. At 200 μg/mL, RhA may inhibited wound closure (p < 0.05), suggesting genuine anti-migratory activity. At 300 μg/mL, stronger inhibition was observed (p < 0.01). However, a contribution from reduced cell viability at this concentration cannot be entirely excluded. The *OLFML2A* gene deletion cohort exhibited a marked reduction in wound closure (Figures 5A,B). We employed the transwell cell invasion experiment, utilising matrigel coating, to assess the invasive capacity of the cells. The findings demonstrated that the suppression of the *OLFML2A* gene may impede the invasion of MDA-MB-231 cells, while the application of RhA also exhibited a notable inhibitory effect on cell invasion. Furthermore, the inhibitory action progressively intensified with the elevation of the RhA administration concentration (Figures 5D,E). The extract may inhibit invasion in a dose-dependent manner, with significant effects observed at 200 μg/mL (*p* < 0.01) and 300 μg/mL (*p* < 0.01). While the effect at 200 μg/mL likely represents genuine anti-invasive activity given the maintained cell viability, interpretation of the 300 μg/mL effect is complicated by concurrent cytotoxicity. We subsequently conducted experimental verification of the cells’ adhesion capability. The findings demonstrated that the cell adhesion capacity was augmented in the presence of a high concentration of RhA (300 μg/mL) (Figures 5C,E). The data demonstrate that silencing *OLFML2A* suppresses the invasion and migration of MDA-MB-231 cells, while RhA may also inhibits these processes in a dose-dependent manner.

### 3.6 RhA mitigates the EMT via OLFML2A in MDA-MB-231 cells

**FIGURE 6:**
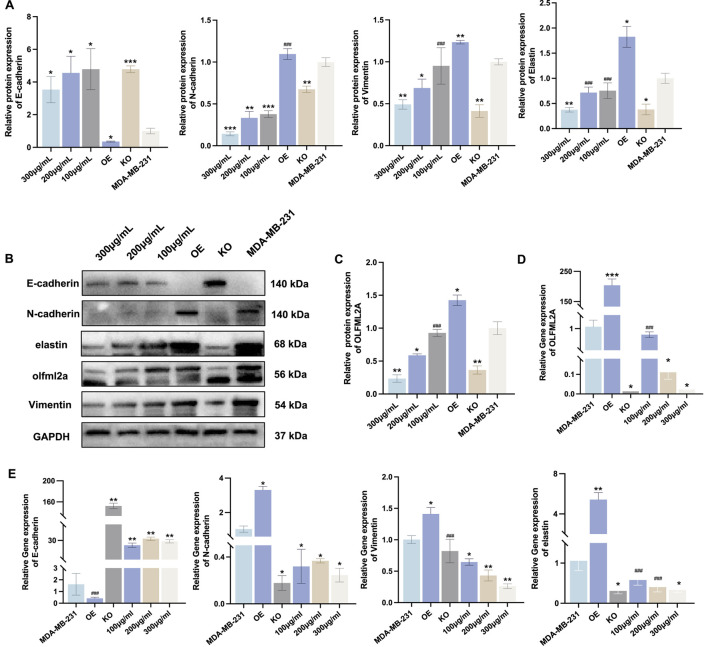
RhA mitigates the EMT via *OLFML2A* in MDA-MB-231 cells. **(A,B)** The comparative protein expression levels of EMT markers (*E-cadherin*, *N-cadherin*, *vimentin*, *elastin*); The expression of protein and mRNA for *OLFML2A* following **(C,D)** treatment; **(E)** mRNA expression of EMT indicators. (***p < 0.001, compared with MDA-MB-231; **p < 0.01, compared with MDA-MB-231; *p < 0.05, compared with MDA-MB-231; ###*p* > 0.05, compared with MDA-MB-231, n = 3).

The role of RhA in inhibiting the EMT process of MDA-MB-231 cells through the suppression of *OLFML2A* expression is currently unclear. To further ascertain the action site of RhA and its impact on the EMT process in MDA-MB-231 cells, we engineered an *OLFML2A* overexpression plasmid to evaluate whether *OLFML2A* overexpression would mitigate the EMT inhibitory effect of RhA and whether the suppression of *OLFML2A* expression would reverse the EMT process. Following the transfection of MDA-MB-231 cells with plasmids that overexpress *OLFML2A*, there was a marked rise in both the mRNA and protein levels of *OLFML2A* (Figures 6C,D). In comparison to wild-type MDA-MB-231 cells, the overexpression of *OLFML2A* diminished the expression of the epithelial marker *E-cadherin*, while concurrently upregulating the mesenchymal markers *N-cadherin*, *vimentin*, and *elastin*, along with the expression of *OLFML2A* (Figures 6A,B,E). In contrast to the *OLFML2A* overexpression group, cells treated with RhA and *OLFML2A* overexpression plasmids exhibited an increase in *E-cadherin*, whereas the levels of mesenchymal markers *N-cadherin*, *vimentin*, and *elastin* diminished, alongside a reduction in *OLFML2A* expression. In comparison to wild-type MDA-MB-231 cells, *E-cadherin* levels in the *OLFML2A* deletion group increased, whereas the expressions of mesenchymal markers *N-cadherin*, *vimentin*, and *elastin* declined, alongside a reduction in *OLFML2A* expression. The data demonstrate that RhA reverses the EMT process via *OLFML2A* in MDA-MB-231 cells.

A central question arising from this study is whether the inhibitory effects of RhA on TNBC cell migration and invasion are directly linked to *OLFML2A* modulation. Our data provide multiple lines of evidence supporting *OLFML2A* as an important mediator of RhA’s biological activity. First, RhA treatment reduced *OLFML2A* expression in a dose-dependent manner. Second, genetic knockdown of *OLFML2A* phenocopied the effects of RhA treatment, producing similar reductions in migration and invasion and similar changes in EMT marker expression (Figures 6A–E).

Several potential mechanisms could explain how RhA modulates *OLFML2A*. RhA might regulate *OLFML2A* transcription through effects on upstream transcription factors such as AP-1, which has been shown to control *OLFML2A* expression in TNBC (Aubin et al., 2022). Alternatively, RhA could influence *OLFML2A* expression indirectly through signaling pathways such as PI3K/AKT, which we have previously shown is modulated by konjac extracts in TNBC cells (Wu et al., 2018), or Wnt/β-catenin, a known regulator of *OLFML2A* in glioma (Ang et al., 2023). Future studies employing chromatin immunoprecipitation, promoter reporter assays, and pathway-specific inhibitors could help elucidate the precise molecular mechanisms by which RhA regulates *OLFML2A* expression.

### 3.7 Major components of RhA identified by LC-MS and GC-MS

**FIGURE 7:**
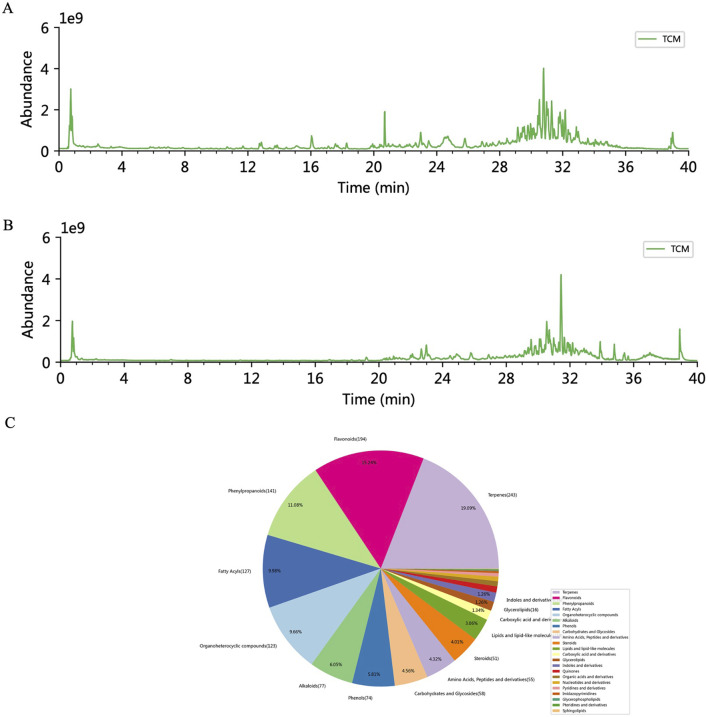
Identification of RhA components. **(A,B)** Total ion current (TIC) chromatograms obtained from LC-MS analysis, representing the sum of all ion intensities or the number of scans within a selected mass range over time. **(C)** Overview of component distribution based on the identification results.

To clarify the major components of RhA, GC-MS analysis was performed. By comparing the resulting spectra with commercial databases, 90 compounds were identified. Following normalization, the detectable constituents of RhA are presented in Table 2, with the major components including 8-(acetyloxy) octyl acetate and undecylenic acid. LC-MS analysis was subsequently employed to detect the components (Figures 7A,B), and the measurable compounds were classified (Figure 7C). These analyses revealed multiple bioactive compounds that overlapped between GC-MS and LC-MS, including undecylenic acid and eicosadienoic acid. Further studies are required to confirm the composition of the petroleum ether extract of *Rhizoma Amorphophalli*.

**TABLE 2:** The main components of RhA determined by GC-MS.

No	RT (min)	Name	Molecular formula	Match factor
1	4.24	8- (Acetyloxy)octyl acetate	C_12_H_22_O_4_	70.31
2	5.53	Nitrous oxide	N_2_O	76.16
3	18.66	1-Butanamine, N,3-dimethyl-	C_6_H_15_N	58.26
4	19.79	Undecylenic acid	C_11_H_20_O_2_	86.40
5	19.86	Phthalic acid, 4-fluoro-2-nitrophenyl methyl ester	C_15_H_10_FNO_6_	87.69
6	21.81	Dodecanoic acid	C_12_H_24_O_2_	92.47
7	22.06	Fumaric acid, ethyl 2-methylallyl ester	C_10_H_14_O_4_	81.80
8	23.28	Tridecanoic acid	C_13_H_26_O_2_	92.72
9	23.98	Pentadecanal-	C_15_H_30_O	95.74
10	24.26	Cyclopropanecarboxylic acid, 2-methyl-2-(4-methyl-3-pentenyl)-, trans- (.+-.)-	C_11_H_18_O_2_	78.29
11	24.39	Myristoleic acid	C_14_H_26_O_2_	93.21
12	24.57	Tetradecanoic acid	C_14_H_28_O_2_	96.54
13	24.95	Tetradecanoic acid, ethyl ester	C_16_H_32_O_2_	90.57
14	25.49	Oxacyclotetradecane-2,11-dione, 13-methyl-	C_14_H_24_O_3_	92.58
15	25.55	Oxacyclotetradecane-2,11-dione, 13-methyl-	C_14_H_24_O_3_	91.91
16	25.64	2,6-Dodecadien-1-al	C_12_H_20_O	83.68
17	25.73	Pentadecanoic acid	C_15_H_30_O_2_	95.86
18	25.85	1,2-Benzenedicarboxylic acid, bis(2-methylpropyl) ester	C_16_H_22_O_4_	94.56
19	26.07	Pentadecanoic acid, ethyl ester	C_17_H_34_O_2_	95.26
20	26.35	Pentadecanal-	C_15_H_30_O	95.07
21	26.51	9,12-Tetradecadien-1-ol, (Z, E)-	C_14_H_26_O	85.30
22	26.60	Palmitoleic acid	C_16_H_30_O_2_	96.66
23	26.69	(E)-Hexadec-9-enoic acid	C_16_H_30_O_2_	87.83
24	26.76	dl-Alanyl-l-alanine	C_6_H_12_N_2_O_3_	70.98
25	26.81	L-Leucine, N-methyl-N- (octyloxycarbonyl)-, dodecyl ester	C_28_H_55_NO_4_	70.27
26	26.81	2H,8H-Benzo[1,2-b:3,4-b']dipyran- 2-one, 8,8-dimethyl-	C_14_H_12_O_3_	59.92
27	26.83	n-Hexadecanoic acid	C_16_H_32_O_2_	96.21
28	26.83	Heptanoic acid, 3,5-dimethyl-, methyl ester	C_10_H_20_O_2_	71.87
29	26.85	1,4-Dibutyl benzene-1,4-dicarboxylate	C_16_H_22_O_4_	85.61
30	26.90	Ethyl 9-hexadecenoate	C_18_H_34_O_2_	91.27
31	27.00	Ethyl 9-hexadecenoate	C_18_H_34_O_2_	77.39
32	27.08	3-(Methylamino)-1,2-propanediol	C_4_H_11_NO_2_	58.02
33	27.11	Hexadecanoic acid, ethyl ester	C_18_H_36_O_2_	95.07
34	27.51	9,12-Octadecadienoic acid (Z, Z)-	C_18_H_32_O_2_	90.77
35	27.56	cis-10-Heptadecenoic acid	C_17_H_32_O_2_	83.02
36	27.78	Heptadecanoic acid	C_17_H_34_O_2_	91.23
37	27.78	cis-3-Methyl-endo-tricyclo [5.2.1.0(2.6)]decane	C_11_H_18_	67.28
38	28.08	Heptadecanoic acid, ethyl ester	C_19_H_38_O_2_	89.15
39	28.34	3-Chloro-N-methylpropylamine	C_4_H_10_ClN	60.27
40	28.37	Pentadecanal-	C_15_H_30_O	94.21
41	28.45	Hexasiloxane, 1,1,3,3,5,5,7,7,9,9,11,11-dodecamethyl-	C_12_H_38_O_5_Si_6_	53.16
42	28.49	Pyrido[2,3-b]isoquinolino[3,4-d] furan-5(6H)-one, 7,9-dimethyl-	C_16_H_12_N_2_OS	66.84
43	28.50	Benzeneacetic acid, .alpha.-amino-4-fluoro-, methyl ester, (.alpha.R)-, Me derivative	C_10_H_12_FNO_2_	61.49
44	28.52	Phenol, 3,4-dimethyl-	C_8_H_10_O	63.17
45	28.52	(9E,11E)-Octadecadienoic acid	C_18_H_32_O_2_	82.92
46	28.54	1-Methyldodecylamine	C_13_H_29_N	58.09
47	28.56	Oleic acid	C_18_H_34_O_2_	88.18
48	28.58	9,12,15-Octadecatrienoic acid, (Z,Z,Z)-	C_18_H_30_O_2_	80.03
49	28.62	sec-Butylamine	C_4_H_11_N	72.78
50	28.65	3,3-Diethoxy-1-propyne	C_7_H_12_O_2_	74.46
51	28.73	9,12-Octadecadienoic acid, ethyl ester	C_20_H_36_O_2_	89.62
52	28.78	(E)-9-Octadecenoic acid ethyl ester	C_20_H_38_O_2_	92.43
53	28.80	2-Hexylpyrazine	C_10_H_16_N_2_	58.91
54	28.80	9,12,15-Octadecatrienoic acid, ethyl ester, (Z,Z,Z)-	C_20_H_34_O_2_	81.19
55	28.92	Dodecanamide	C_12_H_25_NO	78.39
56	29.00	Octadecanoic acid, ethyl ester	C_20_H_40_O_2_	89.71
57	29.39	11,14-Eicosadienoic acid	C_20_H_36_O_2_	88.92
58	29.82	9,12,15-Octadecatrienoic acid, (Z,Z,Z)-	C_18_H_30_O_2_	78.68
59	29.92	3-Cyclopentylpropionic acid, 2-dimethylaminoethyl ester	C_12_H_23_NO_2_	87.71
60	29.97	.beta.-Ocimene	C_10_H_16_	62.33
61	30.04	Glycidyl palmitate	C_19_H_36_O_3_	66.95
62	30.04	Norbornane, 2-isobutyl-	C_11_H_20_	82.77
63	30.07	Piperidine, 1,1′-methylenebis-	C_11_H_22_N_2_	53.15
64	30.17	N-(5-methyl-isoxazol-3-yl)-3- piperidin-1-yl-propionamide	C_12_H_19_N_3_O_2_	69.26
65	30.23	2-((8Z,11Z)-heptadeca-8,11- dien-1-yl)-4,5-dihydrooxazole	C_20_H_35_NO	93.91
66	30.29	Oxazole, 2-(8Z)-8-heptadecen-1-yl-4,5-dihydro-	C_20_H_37_NO	82.90
67	30.30	3-Carene	C_10_H_16_	66.25
68	30.36	1,19-Eicosadiene	C_20_H_38_	92.18
69	30.46	11,14-Eicosadienoic acid	C_20_H_36_O_2_	94.55
70	30.52	Cyclohexadecane, 1,2-diethyl-	C_20_H_40_	70.04
71	30.77	9-Octadecenamide, (Z)-	C_18_H_35_NO	55.27
72	30.77	9,12-Octadecadienoic acid (Z,Z)-	C_18_H_32_O_2_	81.69
73	30.78	Eicosanoic acid	C_20_H_40_O_2_	80.07
74	31.27	9,12-Octadecadien-1-ol, (Z,Z)-	C_18_H_34_O	85.11
75	31.65	Pentadecanal-	C_15_H_30_O	85.96
76	32.08	Carbonic acid, 2-dimethylaminoethyl ethyl ester	C_7_H_15_NO_3_	75.39
77	32.14	Acetic acid, (dodecahydro-7-hydroxy-1,4b, 8,8-tetramethyl-10-oxo-2(1H)-phenanthrenylidene)-,2-(dimethylamino)ethyl ester	C_24_H_39_NO_4_	91.36
78	32.38	Hex-4-yn-3-one	C_6_H_8_O	58.62
79	32.39	4-Bromo-2-methoxybut-2-enoic acid, methyl ester	C_6_H_9_BrO_3_	57.18
80	32.41	Cyclo(glycyl-L-tryptophan), Ac derivative	C_15_H_15_N_3_O_3_	57.16
81	32.41	9-Octadecenoic acid (Z)-, oxiranylmethyl ester	C_21_H_38_O_3_	58.13
82	32.47	10,12-Octadecadienoic acid, 9-oxo-	C_18_H_30_O_3_	63.95
83	32.69	1H-indene, 1-hexadecyl-2,3-dihydro-	C_25_H_42_	58.09
84	32.80	9,12-Octadecadien-1-ol, (Z,Z)-	C_18_H_34_O	83.60
85	33.28	Pentadecanal-	C_15_H_30_O	92.20
86	33.42	cis-13,16-Docasadienoic acid	C_22_H_40_O_2_	94.75
87	33.96	cis-13,16-Docasadienoic acid, methyl ester	C_23_H_42_O_2_	86.89
88	34.33	Nitrous oxide	N_2_O	63.28
89	34.76	11,14-Eicosadienoic acid	C_20_H_36_O_2_	79.90
90	35.87	1-Cyclohexyldimethylsilyloxybutane	C_12_H_26_OSi	62.52

## 4 Discussion

**FIGURE 8:**
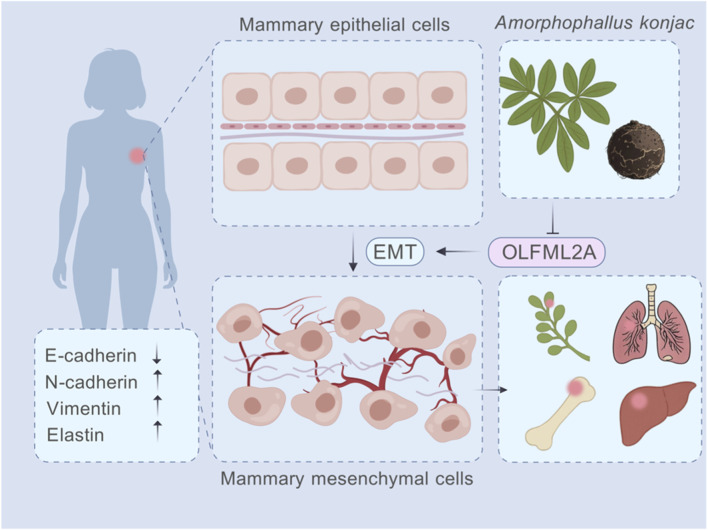
RhA mediates EMT to inhibit the invasion and migration of TNBC cells through *OLFML2A*.

EMT is a multifaceted process, governed by numerous genes, contributing to tumour progression and metastasis (Ang et al., 2023), and is intricately associated with drug resistance in cancer therapy (Xu Z. et al., 2022). In glioblastoma, EMT is modulated by HDAC1, facilitating the invasion and migration of cancer cells (Cheng et al., 2023). In human colorectal cancer (CRC), research indicates that the G protein-coupled receptor μ-opioid receptor (MOR) facilitates EMT via PI3K/AKT signalling, enhances lymph node metastasis, and results in a decreased survival probability among CRC patients (Gao L. et al., 2022). Liu et al. discovered that the FUS/circEZH2/KLF5 feedback loop facilitates CXCR4-induced liver metastasis in breast cancer by augmenting EMT (Liu et al., 2022). USP41 modulates the ubiquitination of Snail, inhibiting its ubiquitination, which leads to enhanced stability of Snail, hence promoting EMT and increasing the migratory capacity of breast cancer cells (Yoon et al., 2023). Consequently, blocking the EMT in tumours may serve as a viable therapeutic strategy to diminish the invasion and migration of breast cancer (Figure 8).

Increasing research suggests that konjac exerts a beneficial therapeutic effect on multiple malignancies. It can counteract tumour invasion and metastasis by upregulating pro-apoptotic proteins, downregulating anti-apoptotic genes, obstructing the cell cycle, increasing apoptosis, limiting proliferation, and diminishing metastasis-associated proteins (Raj et al., 2022; Hu et al., 2025). The study conducted by Sakunie Sawai et al. shown that treatment with konjac glucosaminoglycan drastically reduced the viability of the human hepatocellular carcinoma cell line HepG2, correlating with the appearance of apoptosis-like cells. The inhibitory effect may be mediated via the Bcl2/Bax protein pathway (Sawai et al., 2018). In a murine model of liver cancer, konjac glucomannan mitigated the multidrug resistance of HepG2/5-FU cells by decreasing AKT signalling and enhancing p53 expression (Chen et al., 2020). Chen et al.'s research demonstrated that konjac enhances apoptosis and induces cell cycle arrest in gastric cancer cells, significantly inhibits the proliferation of gastric cancer cell lines SGC-7901 and AGS, and improves the quality of life for gastric cancer patients *in vivo*, exhibiting potential anti-tumor activity (Chen et al., 2017). Munmi Majumder et al. discovered that konjac extract prompted apoptosis in breast cancer cells and reduced cell invasion, migration, and adhesion, particularly in MDA-MB-231 cells, demonstrating a pronounced effect in inhibiting adhesion and invasion (Majumder et al., 2021). Mei et al. identified by network pharmacology, molecular linkage, and experimental validation that Scutellarein and genistein in the ethyl konjac acetate extract exhibit therapeutic promise against TNBC by targeting EGFR and ESR1 while blocking the PI3K/Akt signalling pathway (Mei et al., 2025). Our team’s prior research indicated that in TNBC, konjac suppresses the proliferation, migration, invasion, and metastasis of TNBC cells via the PI3K/AKT/mTOR pathway (Wu et al., 2018). This study revealed that RhA greatly enhances the expression of *E-cadherin* while diminishing the expressions of *OLFML2A*, *N-cadherin*, *vimentin*, and *elastin*. Furthermore, RhA may substantially impede the invasion and migratory capabilities of MDA-MB-231 cells. This suggests that RhA exerts a considerable inhibitory influence on the EMT process in TNBC. Moreover, the inhibitory effect of RhA on EMT in TNBC demonstrates a notable dose-dependent relationship. This investigation revealed that with increasing concentrations of RhA, the suppression of EMT grew more pronounced, including a marked reduction in the invasion and migratory capabilities of the cells. The dosage of RhA utilised in this work differs from that employed in other research, suggesting that the MDA-MB-231 cell line exhibits varying sensitivity to different quantities of RhA. This study is the first to report that RhA may suppress EMT in breast cancer.

Increasing evidence suggests a link between *OLFML2A* and the incidence of human tumours. Ma et al. discovered that the expression of *OLFML2A* was elevated in glioma specimens and had a favourable correlation with the pathological grade of glioma patients. Inhibition of *OLFML2A* suppresses Wnt/β-catenin signalling by enhancing the expression of amyloid precursor protein (APP) and diminishing the stable level of β-catenin (Ma et al., 2021). Research by Sun et al. indicated that elevated levels of *OLFML2A* may correlate with unfavourable outcomes in acute myeloid leukaemia (AML) (Sun et al., 2023) and contribute to the extramedullary invasion associated with the disease (Lv et al., 2018). Bai et al. established via weighted gene co-expression network analysis (WGCNA) that *OLFML2A* negatively regulates angiogenesis, DNA-binding transcription factor activity, apoptosis, and autophagy, and serves as a potential therapeutic target for preserving the stemness of hepatocellular carcinoma cells (Bai et al., 2020). *OLFML2A* frequently plays a significant role in breast cancer. Zhao et al. discovered that in patients with TNBC, the expression of the activator protein-1 (AP-1) family was markedly elevated, and *OLFML2A* serves as a crucial regulatory protein downstream of AP-1, being essential for TNBC characterised by AP-1 overexpression (Zhao et al., 2021). Our team’s prior study identified *OLFML2A* as a possible therapeutic target for TNBC. Inhibition of the *OLFML2A* gene can trigger apoptosis by facilitating S-phase arrest in TNBC cells. The overexpression of *OLFML2A* may enhance proliferation, migration, and invasion associated with TNBC (Gao X. et al., 2022). This work reveals that *OLFML2A* is associated with the EMT process in TNBC through the examination of cancer tissues, surrounding tissue samples, and cellular studies with TNBC patients. High expression of *OLFML2A* dramatically downregulates the epithelial marker *E-cadherin*. The expressions of mesenchymal markers *N-cadherin*, *vimentin*, and *elastin* were each upregulated to varying degrees in the same specimens. In the *OLFML2A*-silenced TNBC cell line MDA-MB-231, the cells’ invasion and migration capabilities were diminished compared to the wild-type MDA-MB-231 cell line, whereas the invasion and migration capabilities of the *OLFML2A*-overexpressing TNBC cell line MDA-MB-231 were significantly enhanced. This work is the inaugural publication that integrates clinical tumour tissues from TNBC patients to examine the influence of *OLFML2A* on EMT.

We observed higher *OLFML2A* expression in MDA-MB-231 cells compared to MDA-MB-453 cells (Figures 3A,B), reflecting TNBC’s molecular heterogeneity. MDA-MB-231 belongs to the mesenchymal stem-like (MSL) subtype, characterized by EMT features and high invasiveness, while MDA-MB-453 is classified as luminal androgen receptor (LAR) subtype with lower invasive potential. The higher *OLFML2A* expression in MDA-MB-231 aligns with its mesenchymal phenotype and our finding that *OLFML2A* promotes EMT. These cell lines also exhibit distinct signaling pathway activation: MDA-MB-231 shows constitutive activation of MEK/ERK and TGFβ/SMAD3 pathways that promote EMT, while MDA-MB-453 relies on β-catenin signaling downstream of androgen receptor (Zhang et al., 2023b; Xu J. et al., 2022; Ahram et al., 2021). Additionally, epigenetic differences such as promoter methylation may contribute to differential *OLFML2A* expression.Functionally, higher *OLFML2A* in MDA-MB-231 correlates with its mesenchymal morphology and invasive capacity, and our knockdown experiments confirmed *OLFML2A*’s causal role in these phenotypes (Figures 4, 5). These observations suggest *OLFML2A* as a subtype-specific driver of TNBC aggressiveness, with particular relevance to mesenchymal-like tumors, informing future precision medicine approaches.

In the present study, we observed that *OLFML2A* expression was significantly upregulated in cancerous tissues compared to adjacent non-cancerous tissues in only 8 out of 18 TNBC patients (44.4%), while the remaining 10 patients exhibited no marked increase (Figures 1C–E). This inter-patient heterogeneity in *OLFML2A* expression raises important questions regarding its role as a universal mediator of TNBC progression and warrants further discussion.

Several factors may account for this variability. First, tumor heterogeneity—a hallmark of TNBC—may underlie differential *OLFML2A* expression. TNBC is known to encompass multiple molecular subtypes, including basal-like 1, basal-like 2, mesenchymal, and luminal androgen receptor subtypes, each with distinct transcriptomic profiles and biological behaviors (Yin et al., 2020). It is plausible that *OLFML2A* expression is enriched in specific TNBC subtypes that exhibit higher metastatic potential or mesenchymal traits. Indeed, our data showed that *OLFML2A*-high tumors were associated with reduced *E-cadherin* and elevated mesenchymal markers (Figure 2), suggesting a possible link between *OLFML2A* expression and a mesenchymal-like phenotype. Second, tumor stage and grade may influence *OLFML2A* levels. Although our sample size limited stratified analysis, it is conceivable that *OLFML2A* expression correlates with advanced disease stages or higher histological grades, as previously reported in glioma (Ma et al., 2021). Thirdly, epigenetic regulation or stromal-tumor interactions within the tumor microenvironment could modulate *OLFML2A* expression independently of genetic alterations. For instance, hypoxia or inflammatory cytokines may induce *OLFML2A* in a subset of tumors, contributing to its heterogeneous expression pattern.

The presence of *OLFML2A*-low tumors does not diminish the biological significance of *OLFML2A* in TNBC progression. Instead, it underscores the concept that *OLFML2A* may function as a context-dependent driver of EMT and metastasis, active only in a subset of TNBC patients with specific molecular or microenvironmental backgrounds. This is consistent with the observation that *OLFML2A*-high tumors displayed clear EMT features, whereas *OLFML2A*-low tumors did not (Supplementary Material). Importantly, *in vitro* experiments demonstrated that *OLFML2A* knockdown consistently suppressed EMT and invasion in MDA-MB-231 cells (a high-*OLFML2A*-expressing line), while overexpression enhanced these phenotypes (Figures 4, 5). These findings support a causal role of *OLFML2A* in promoting TNBC aggressiveness, even if its upregulation is not universal across all patients. From a translational perspective, the heterogeneity of *OLFML2A* expression suggests that patient stratification based on *OLFML2A* status may be necessary for future therapeutic strategies targeting this gene. *OLFML2A* could serve as a biomarker for identifying TNBC patients at higher risk of metastasis who might benefit from *OLFML2A*-targeted interventions, such as konjac-derived extracts or other inhibitors. Future studies with larger cohorts and comprehensive clinicopathological annotation are warranted to validate these associations and elucidate the mechanisms underlying *OLFML2A* regulation in TNBC.

This study revealed moderate but significant correlations between *OLFML2A* and EMT markers in TNBC tissues (Figure 2E). While statistically significant, these moderate correlations indicate that *OLFML2A* expression partially explains the variance in EMT marker expression, suggesting additional factors contribute to EMT regulation in TNBC. Several explanations may account for this. First, *OLFML2A* may regulate EMT indirectly via intermediate signaling pathways such as Wnt/β-catenin rather than directly controlling EMT gene transcription (Ma et al., 2021). Secondly, EMT is regulated by multiple transcription factors and pathways. For instance, transforming growth factor β (TGFβ) mediates EMT through diverse signal cascades, regulating the sequential expression waves of various other cytokines and promoting the gradual remodeling of the extracellular matrix that facilitates basement membrane movement (Tsubakihara and Moustakas, 2018). Pro-inflammatory cytokine TNF-α can induce the expression of mesenchymal genes in patient-derived cell models (Aubin et al., 2022). And *OLFML2A* is likely to be a metabolites of this larger network. Thirdly, tumor heterogeneity and microenvironmental factors like hypoxia, inflammation may modulate the *OLFML2A*-EMT relationship in a context-dependent manner. Fourth, post-transcriptional regulation of EMT markers could weaken correlations with *OLFML2A* mRNA levels. Thus, *OLFML2A* likely functions as one of multiple EMT regulators within a complex network, rather than a solitary master switch.

In summary, this study provides evidence that konjac petroleum ether extract exerts anti-migratory and anti-invasive effects on TNBC cells through *OLFML2A*-mediated suppression of EMT. While the findings are promising, they should be interpreted in light of the limitations outlined above. Future investigations addressing these gaps will be critical to advancing this botanical extract toward potential clinical application.

A central question arising from this study is whether the inhibitory effects of RhA on TNBC cells are mediated exclusively through *OLFML2A* or whether additional pathways contribute. Several lines of evidence support *OLFML2A* as a key mediator: RhA treatment significantly reduced *OLFML2A* expression; *OLFML2A* knockdown phenocopied RhA effects on migration, invasion, and EMT markers; and *OLFML2A* overexpression partially rescued cells from RhA-induced inhibition. However, the rescue was only partial, and the moderate correlation between *OLFML2A* and EMT markers in patient tissues suggests that *OLFML2A* explains only part of the variance in EMT phenotype. These observations, together with the complex polypharmacological nature of botanical extracts, indicate that RhA likely exerts its effects through both *OLFML2A*-dependent and *OLFML2A*-independent mechanisms. Candidate pathways that may contribute independently of *OLFML2A* include PI3K/AKT, which our previous work implicated in konjac-mediated TNBC inhibition, as well as Wnt/β-catenin and TGF-β signaling, both known to regulate EMT. Future studies employing pathway-specific inhibitors and unbiased omics approaches will be necessary to fully delineate the relative contributions of these parallel mechanisms.

In conclusion, RhA can partly diminish the expression of *OLFML2A* to impede the EMT of TNBC. RhA may have potential as an EMT inhibitor for further investigation in TNBC and possesses significant therapeutic promise though additional studies are required.

## 5 Limitation and perspective

This study suggests that konjac petroleum ether extract may inhibit migration and invasion of TNBC cells, at least in part through suppression of *OLFML2A*-mediated EMT. Using clinical tissue specimens, we observed that *OLFML2A* expression is elevated in a subset of TNBC patients and correlates with reduced *E-cadherin* and increased mesenchymal markers (*N-cadherin*, *vimentin*, *elastin*), suggesting a role for *OLFML2A* in EMT regulation. *In vitro* experiments confirmed that *OLFML2A* knockdown phenocopied the effects of the extract, reducing cell migration and invasion while reversing EMT marker expression. However, several limitations of this study must be acknowledged. First, the clinical sample size (n = 18) is relatively small, and the observed heterogeneity in *OLFML2A* expression—with only 8 of 18 patients showing significant upregulation—requires validation in larger, multi-center cohorts with comprehensive clinicopathological annotation. Second, while we infer selectivity for cancer cells based on differential *OLFML2A* expression and prior literature direct cytotoxicity data on normal mammary epithelial cells (e.g., MCF-10A) using this specific extract are lacking. Such experiments are essential to definitively establish a therapeutic window. Third, the absence of time-dependent cytotoxicity studies limits our understanding of whether the effects observed at higher concentrations (particularly 300 μg/mL) represent genuine anti-migratory activity or are confounded by reduced cell viability. Fourth, this study is entirely *in vitro*, and the translational relevance of our findings remains to be validated in appropriate *in vivo* models (e.g., orthotopic xenograft mouse models of TNBC metastasis). Fifth, the mechanistic depth is incomplete; while we have identified *OLFML2A* as a key mediator, the upstream regulators of *OLFML2A* and its downstream effectors beyond the EMT markers examined here warrant further investigation.

Besides this, the distinction between “therapeutic” and “toxic” depends critically on selectivity for cancer cells over normal cells. While direct normal cell cytotoxicity data for this specific extract are not presented in our research, previous studies have demonstrated that konjac extracts exhibit selective anticancer activity (Jain et al., 2025; Hu et al., 2025). Wu et al. reported that konjac extract at 200 μg/mL reduced MDA-MB-231 viability while maintaining MCF-10A viability (Li et al., 2022; Wu et al., 2018). Furthermore, the differential expression of *OLFML2A* between cancer and normal cells (Figures 3A,B) provides a mechanistic basis for this potential selectivity, as our study suggests that the extract’s effects are partially mediated through *OLFML2A*. We acknowledge that the absence of time-dependent studies and direct normal cell cytotoxicity data are limitations. Future studies should include kinetic analyses and systematic selectivity evaluation to fully establish the therapeutic window.

Besides, functional assays (wound healing, invasion) were performed only on TNBC cells, not on normal mammary epithelial cells. While previous studies have suggested that konjac extracts minimally affect normal breast cell migration and the differential expression of *OLFML2A* between cancer and normal cells (Figures 3A,B) provides a mechanistic basis for selectivity, direct experimental validation using this specific extract is lacking. Future studies should include parallel functional assays on normal cells to definitively establish the selectivity of the observed anti-migratory and anti-invasive effects.

Building on these findings, several future directions are warranted. First, phytochemical fractionation and metabolomic profiling should be pursued to identify the specific active constituents responsible for the observed biological activity. Second, kinetic analyses across multiple time points (e.g., 24, 48, 72 h) and systematic selectivity evaluation using normal cell lines should be performed to fully characterize the extract’s therapeutic window. Third, mechanistic studies employing transcriptomics, proteomics, or chromatin immunoprecipitation could elucidate the broader regulatory network in which *OLFML2A* operates and identify direct vs. indirect targets. Fourth, *in vivo* validation using TNBC metastasis models is essential to determine whether the anti-migratory and anti-invasive effects observed *in vitro* translate to reduced metastatic burden in living organisms. Fifth, given the heterogeneity of *OLFML2A* expression across TNBC patients, future studies should explore whether *OLFML2A* expression status can serve as a predictive biomarker for patient stratification, identifying those most likely to benefit from konjac-based or *OLFML2A*-targeted therapies.

## 6 Conclusion

This study suggests that RhA inhibits migration and invasion of TNBC cells, with *OLFML2A* serving as a key mediator of these effects. Using clinical tissue specimens from 18 TNBC patients, we observed that *OLFML2A* expression is elevated in a subset of patients and correlates with reduced *E-cadherin* and increased mesenchymal markers including *N-cadherin*, *vimentin*, and *elastin*, suggesting a role for *OLFML2A* in EMT regulation. In contrast, patients with low *OLFML2A* expression showed no significant EMT marker alterations (Supplementary Material), confirming that the EMT phenotype is specifically associated with high *OLFML2A* expression. *In vitro* experiments confirmed that genetic knockdown of *OLFML2A* phenocopied the effects of RhA treatment, reducing migration and invasion while reversing EMT marker expression. RhA treatment alone inhibited cell proliferation with an IC_50_ of 220 μg/mL at 48 h.

In summary, this study provides evidence that konjac petroleum ether extract exerts anti-migratory and anti-invasive effects on TNBC cells through *OLFML2A*-mediated suppression of EMT, with an IC_50_ of 220 μg/mL and inhibition of migration and invasion at 200 μg/mL. While the findings are promising, they should be interpreted in light of the limitations outlined above. Future investigations addressing these gaps will be critical to advancing this botanical extract toward potential clinical application, particularly for TNBC patients with high *OLFML2A* expression.

## Data Availability

The original contributions presented in the study are publicly available. This data can be found here: https://doi.org/10.6084/m9.figshare.32210214; https://doi.org/10.6084/m9.figshare.32203299.
