# Supplementary material for: Konjac petroleum ether extract inhibits triple-negative breast cancer cell migration and invasion by attenuating OLFML2A-mediated epithelial-mesenchymal transition
Source: Front Pharmacol. 2026 May 15;17:1734640. doi: 10.3389/fphar.2026.1734640 (PMC13219017; doi:10.3389/fphar.2026.1734640)

Supplementary Table S1 LC-MS analysis of *Konjac* petroleum ether extract. The table shows the major of chemical compounds isolated from the *Konjac* petroleum ether extract.

No.	Structure	Name	Molecular Formula
1	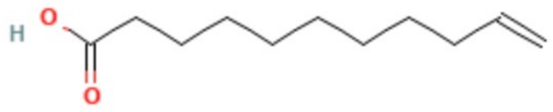	Undecenoic acid	$C_{11}H_{20}O_2$
2	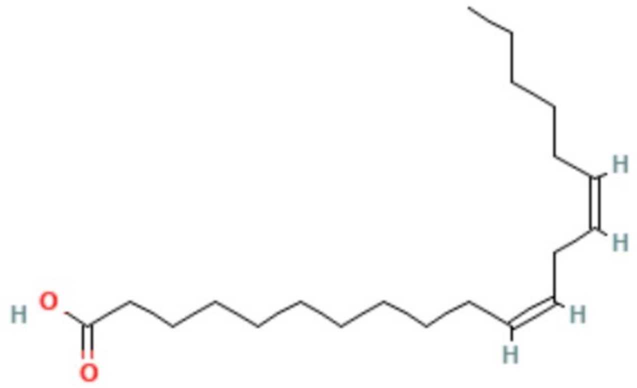	Eicosadienoic acid	$C_{20}H_{36}O_2$
3	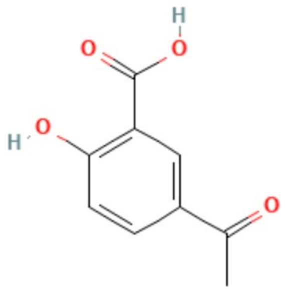	5-Acetylsalicylic acid	$C_9H_8O_4$

**4**	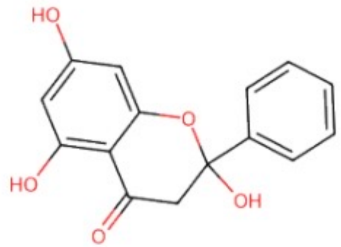	2-hydroxypinocembrin	$C_{15}H_{12}O_5$
**5**	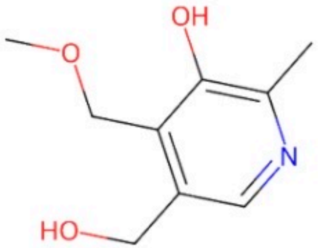	4’-O-Methylpyridoxine	$C_9H_{13}NO_3$
**6**	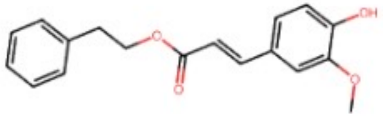	Phenethyl ferulate	$C_{18}H_{18}O_4$
**7**	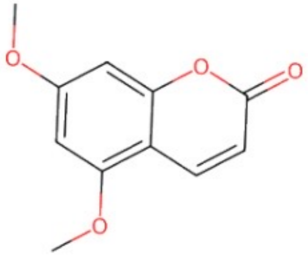	Citropten	$C_{11}H_{10}O_4$

*Images of the chemical structures of the bioactive compounds in *Konjac* ether extract were obtained from the PubChem database.

Supplementary Figure S1 LC-MS analysis of *Konjac* petroleum ether extract. The figure shows the TIC (Total Ion Chromatogram) graph for the sum of all ion intensities over time or the number of scans within the selected quality range.

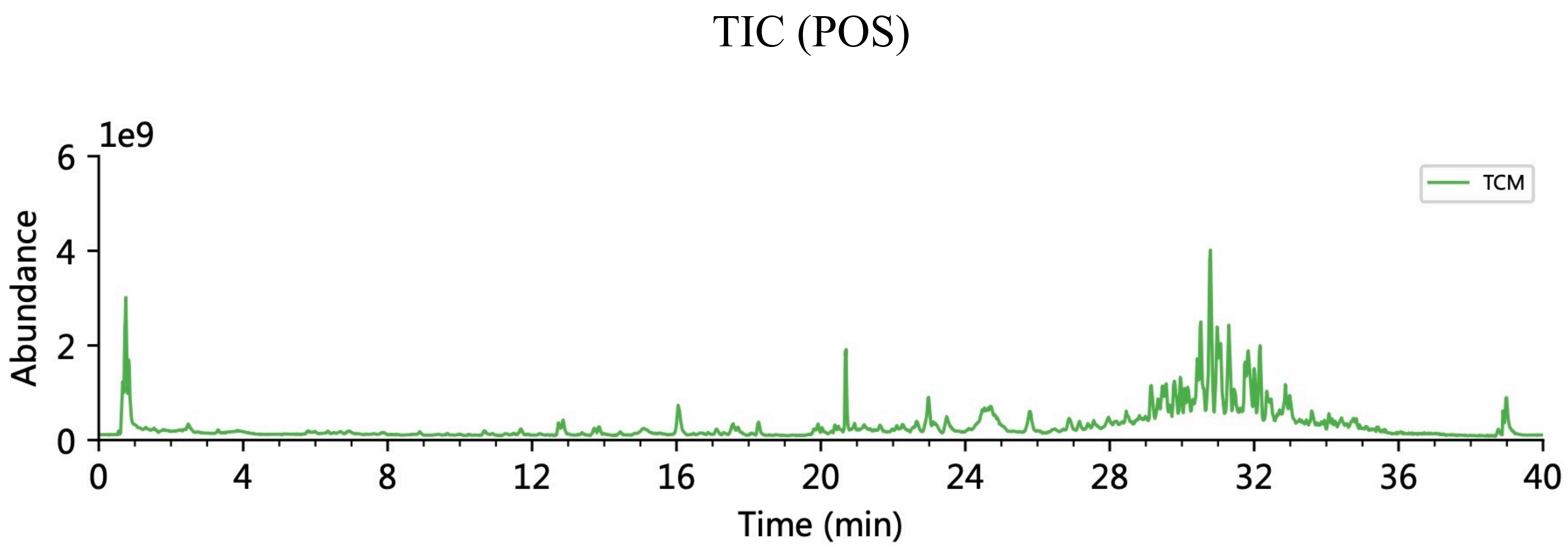

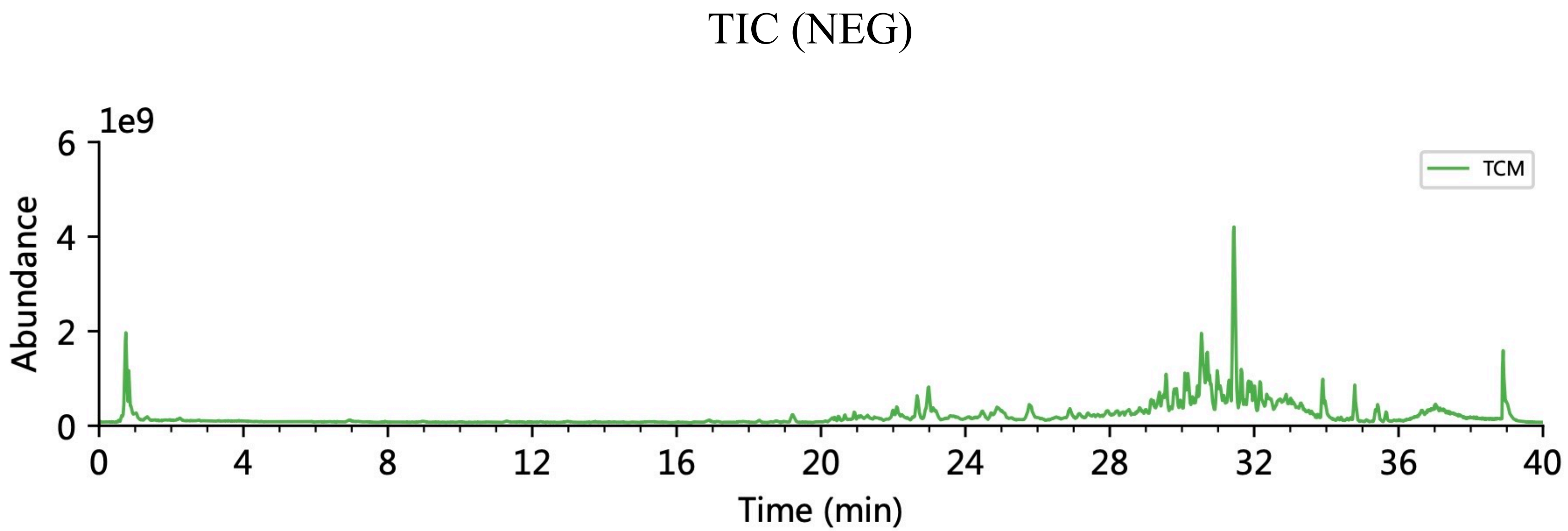

Supplementary Figure S2 LC-MS analysis of *Konjac* petroleum ether extract. The figure shows a qualitative distribution map of components based on the results of component identification.

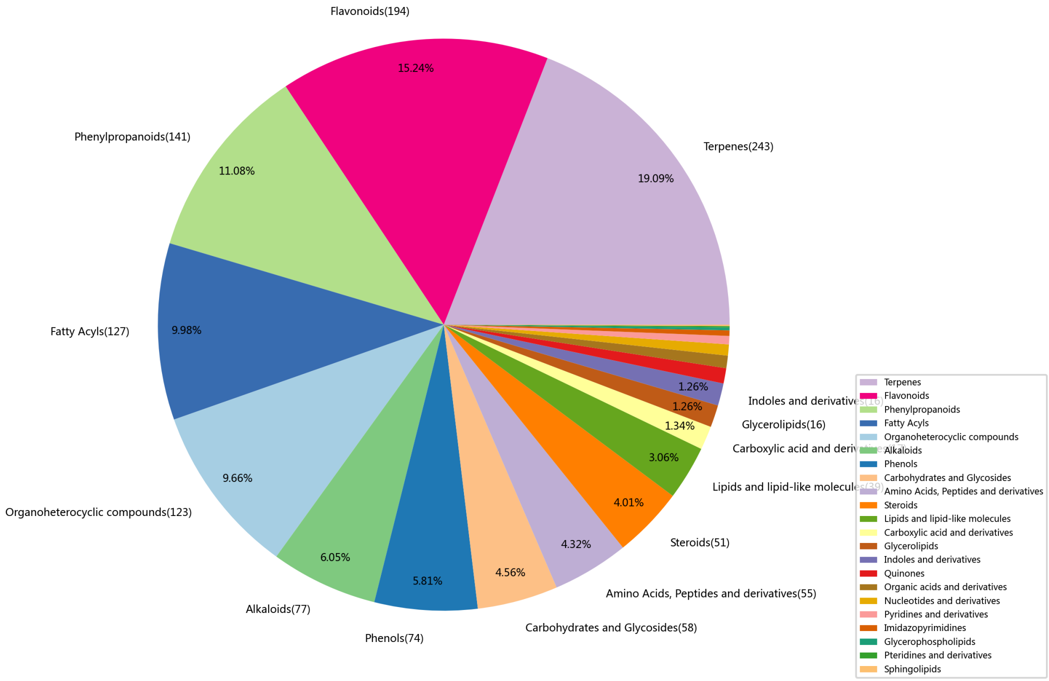

For traditional Chinese medicine samples, approximately 100 mg of each sample was extracted with 1 mL of 70% methanol in water containing the same internal standard, followed by grinding at 60 Hz for 2 min, ultrasonic extraction in an ice-water bath for 60 min, and centrifugation at 12,000 rpm for 10 min at 4 °C; 200 μL of the supernatant was then collected for analysis. Chromatographic separation was performed on an ACQUITY UPLC HSS T3 column (100 mm × 2.1 mm, 1.8 μm) at 45 °C, with a mobile phase consisting of water (containing 0.1% formic acid) and acetonitrile at a flow rate of 0.35 mL/min and an injection volume of 2 μL. Mass spectrometry was conducted using a heated electrospray ionization source in both positive and negative ion modes, with data acquisition performed in data-dependent acquisition mode (Full MS/dd-MS^2, TOP 10).

Supplementary Table S2 GC-MS analysis of *Konjac* petroleum ether extract. The table shows the major of chemical compounds isolated from the *Konjac* petroleum ether extract. (Chunyu Wu et al., 2018)

No	RT (min)	Name	Molecular Formula	Match Factor
**1**	4.24	8-(Acetyloxy)octyl acetate	$C_{12}H_{22}O_4$	70.31
**2**	5.53	Nitrous oxide	N_2O	76.16
**3**	18.66	1-Butanamine, N,3-dimethyl-	$C_6H_{15}N$	58.26
**4**	19.79	Undecylenic acid	$C_{11}H_{20}O_2$	86.40
**5**	19.86	Phthalic acid, 4-fluoro-2-nitrophenyl methyl ester	$C_{15}H_{10}FNO_6$	87.69
**6**	21.81	Dodecanoic acid	$C_{12}H_{24}O_2$	92.47
**7**	22.06	Fumaric acid, ethyl 2-methylallyl ester	$C_{10}H_{14}O_4$	81.80

Supplementary Table S2 GC-MS analysis of *Konjac* petroleum ether extract. The table shows the major of chemical compounds isolated from the *Konjac* petroleum ether extract. (Chunyu Wu et al., 2018)

No	RT (min)	Name	Molecular Formula	Match Factor
**8**	23.28	Tridecanoic acid	C13H26O2	92.72
**9**	23.98	Pentadecanal-	C15H30O	95.74
**10**	24.26	Cyclopropanecarboxylic acid, 2-methyl-2-(4-methyl-3-pentenyl)-, trans-(.+-.)-	C11H18O2	78.29
**11**	24.39	Myristoleic acid	C14H26O2	93.21
**12**	24.57	Tetradecanoic acid	C14H28O2	96.54
**13**	24.95	Tetradecanoic acid, ethyl ester	C16H32O2	90.57
**14**	25.49	Oxacyclotetradecane-2,11-dione, 13-methyl-	C14H24O3	92.58

Supplementary Table S2 GC-MS analysis of *Konjac* petroleum ether extract. The table shows the major of chemical compounds isolated from the *Konjac* petroleum ether extract. (Chunyu Wu et al., 2018)

No	RT (min)	Name	Molecular Formula	Match Factor
**15**	25.55	Oxacyclotetradecane-2,11-dione, 13-methyl-	$C_{14}H_{24}O_3$	91.91
**16**	25.64	2,6-Dodecadien-1-al	$C_{12}H_{20}O$	83.68
**17**	25.73	Pentadecanoic acid	$C_{15}H_{30}O_2$	95.86
**18**	25.85	1,2-Benzenedicarboxylic acid, bis(2-methylpropyl) ester	$C_{16}H_{22}O_4$	94.56
**19**	26.07	Pentadecanoic acid, ethyl ester	$C_{17}H_{34}O_2$	95.26
**20**	26.35	Pentadecanal-	$C_{15}H_{30}O$	95.07
**21**	26.51	9,12-Tetradecadien-1-ol, (Z,E)-	$C_{14}H_{26}O$	85.30

Supplementary Table S2 GC-MS analysis of *Konjac* petroleum ether extract. The table shows the major of chemical compounds isolated from the *Konjac* petroleum ether extract. (Chunyu Wu et al., 2018)

No	RT (min)	Name	Molecular Formula	Match Factor
**22**	26.60	Palmitoleic acid	$C_{16}H_{30}O_2$	96.66
**23**	26.69	(E)-Hexadec-9-enoic acid	$C_{16}H_{30}O_2$	87.83
**24**	26.76	dl-Alanyl-l-alanine	$C_6H_{12}N_2O_3$	70.98
**25**	26.81	L-Leucine, N-methyl-N-(octyloxycarbonyl)-, dodecyl ester	$C_{28}H_{55}NO_4$	70.27
**26**	26.81	2H,8H-Benzo[1,2-b:3,4-b']dipyran-2-one, 8,8-dimethyl-	$C_{14}H_{12}O_3$	59.92
**27**	26.83	n-Hexadecanoic acid	$C_{16}H_{32}O_2$	96.21
**28**	26.83	Heptanoic acid, 3,5-dimethyl-, methyl ester	$C_{10}H_{20}O_2$	71.87

Supplementary Table S2 GC-MS analysis of *Konjac* petroleum ether extract. The table shows the major of chemical compounds isolated from the *Konjac* petroleum ether extract. (Chunyu Wu et al., 2018)

No	RT (min)	Name	Molecular Formula	Match Factor
**29**	26.85	1,4-Dibutyl benzene-1,4-dicarboxylate	C16H22O4	85.61
**30**	26.90	Ethyl 9-hexadecenoate	C18H34O2	91.27
**31**	27.00	Ethyl 9-hexadecenoate	C18H34O2	77.39
**32**	27.08	3-(Methylamino)-1,2-propanediol	C4H11NO2	58.02
**33**	27.11	Hexadecanoic acid, ethyl ester	C18H36O2	95.07
**34**	27.51	9,12-Octadecadienoic acid (Z,Z)-	C18H32O2	90.77
**35**	27.56	cis-10-Heptadecenoic acid	C17H32O2	83.02

Supplementary Table S2 GC-MS analysis of *Konjac* petroleum ether extract. The table shows the major of chemical compounds isolated from the *Konjac* petroleum ether extract. (Chunyu Wu et al., 2018)

No	RT (min)	Name	Molecular Formula	Match Factor
**36**	27.78	Heptadecanoic acid	$C_{17}H_{34}O_2$	91.23
**37**	27.78	cis-3-Methyl-endo-tricyclo[5.2.1.0(2.6)]decane	$C_{11}H_{18}$	67.28
**38**	28.08	Heptadecanoic acid, ethyl ester	$C_{19}H_{38}O_2$	89.15
**39**	28.34	3-Chloro-N-methylpropylamine	$C_4H_{10}ClN$	60.27
**40**	28.37	Pentadecanal-	$C_{15}H_{30}O$	94.21
**41**	28.45	Hexasiloxane, 1,1,3,3,5,5,7,7,9,9,11,11-dodecamethyl-	$C_{12}H_{38}O_5Si_6$	53.16
**42**	28.49	Pyrido[2,3-b]isoquinolino[3,4-d] furan-5(6H)-one, 7,9-dimethyl-	$C_{16}H_{12}N_2OS$	66.84

Supplementary Table S2 GC-MS analysis of *Konjac* petroleum ether extract. The table shows the major of chemical compounds isolated from the *Konjac* petroleum ether extract. (Chunyu Wu et al., 2018)

No	RT (min)	Name	Molecular Formula	Match Factor
**43**	28.50	Benzeneacetic acid, .alpha.-amino-4-fluoro-, methyl ester, (.alpha.R)-, Me derivative	C10H12FNO2	61.49
**44**	28.52	Phenol, 3,4-dimethyl-	C8H10O	63.17
**45**	28.52	(9E,11E)-Octadecadienoic acid	C18H32O2	82.92
**46**	28.54	1-Methyldodecylamine	C13H29N	58.09
**47**	28.56	Oleic Acid	C18H34O2	88.18
**48**	28.58	9,12,15-Octadecatrienoic acid, (Z,Z,Z)-	C18H30O2	80.03
**49**	28.62	sec-Butylamine	C4H11N	72.78

Supplementary Table S2 GC-MS analysis of *Konjac* petroleum ether extract. The table shows the major of chemical compounds isolated from the *Konjac* petroleum ether extract. (Chunyu Wu et al., 2018)

No	RT (min)	Name	Molecular Formula	Match Factor
**50**	28.65	3,3-Diethoxy-1-propyne	$C_7H_{12}O_2$	74.46
**51**	28.73	9,12-Octadecadienoic acid, ethyl ester	$C_{20}H_{36}O_2$	89.62
**52**	28.78	(E)-9-Octadecenoic acid ethyl ester	$C_{20}H_{38}O_2$	92.43
**53**	28.80	2-Hexylpyrazine	$C_{10}H_{16}N_2$	58.91
**54**	28.80	9,12,15-Octadecatrienoic acid, ethyl ester, (Z,Z,Z)-	$C_{20}H_{34}O_2$	81.19
**55**	28.92	Dodecanamide	$C_{12}H_{25}NO$	78.39
**56**	29.00	Octadecanoic acid, ethyl ester	$C_{20}H_{40}O_2$	89.71

Supplementary Table S2 GC-MS analysis of *Konjac* petroleum ether extract. The table shows the major of chemical compounds isolated from the *Konjac* petroleum ether extract. (Chunyu Wu et al., 2018)

No	RT (min)	Name	Molecular Formula	Match Factor
**57**	29.39	11,14-Eicosadienoic acid	$C_{20}H_{36}O_2$	88.92
**58**	29.82	9,12,15-Octadecatrienoic acid, (Z,Z,Z)-	$C_{18}H_{30}O_2$	78.68
**59**	29.92	3-Cyclopentylpropionic acid, 2-dimethylaminoethyl ester	$C_{12}H_{23}NO_2$	87.71
**60**	29.97	.beta.-Ocimene	$C_{10}H_{16}$	62.33
**61**	30.04	Glycidyl palmitate	$C_{19}H_{36}O_3$	66.95
**62**	30.04	Norbornane, 2-isobutyl-	$C_{11}H_{20}$	82.77
**63**	30.07	Piperidine, 1,1'-methylenebis-	$C_{11}H_{22}N_2$	53.15

Supplementary Table S2 GC-MS analysis of *Konjac* petroleum ether extract. The table shows the major of chemical compounds isolated from the *Konjac* petroleum ether extract. (Chunyu Wu et al., 2018)

No	RT (min)	Name	Molecular Formula	Match Factor
**64**	30.17	N-(5-Methyl-isoxazol-3-yl)-3-piperidin-1-yl-propionamide	$C_{12}H_{19}N_3O_2$	69.26
**65**	30.23	2-((8Z,11Z)-Heptadeca-8,11-dien-1-yl)-4,5-dihydrooxazole	$C_{20}H_{35}NO$	93.91
**66**	30.29	Oxazole, 2-(8Z)-8-heptadecen-1-yl-4,5-dihydro-	$C_{20}H_{37}NO$	82.90
**67**	30.30	3-Carene	$C_{10}H_{16}$	66.25
**68**	30.36	1,19-Eicosadiene	$C_{20}H_{38}$	92.18
**69**	30.46	11,14-Eicosadienoic acid	$C_{20}H_{36}O_2$	94.55
**70**	30.52	Cyclohexadecane, 1,2-diethyl-	$C_{20}H_{40}$	70.04

Supplementary Table S2 GC-MS analysis of *Konjac* petroleum ether extract. The table shows the major of chemical compounds isolated from the *Konjac* petroleum ether extract. (Chunyu Wu et al., 2018)

No	RT (min)	Name	Molecular Formula	Match Factor
**71**	30.77	9-Octadecenamide, (Z)-	C18H35NO	55.27
**72**	30.77	9,12-Octadecadienoic acid (Z,Z)-	C18H32O2	81.69
**73**	30.78	Eicosanoic acid	C20H40O2	80.07
**74**	31.27	9,12-Octadecadien-1-ol, (Z,Z)-	C18H34O	85.11
**75**	31.65	Pentadecanal-	C15H30O	85.96
**76**	32.08	Carbonic acid, 2-dimethylaminoethyl ethyl ester	C7H15NO3	75.39
**77**	32.14	Acetic acid, (dodecahydro-7-hydroxy-1,4b, 8,8-tetramethyl-10-oxo-2(1H)-phenanthrenylidene)- ,2-(dimethylamino)ethyl ester	C24H39NO4	91.36

Supplementary Table S2 GC-MS analysis of *Konjac* petroleum ether extract. The table shows the major of chemical compounds isolated from the *Konjac* petroleum ether extract. (Chunyu Wu et al., 2018)

No	RT (min)	Name	Molecular Formula	Match Factor
**78**	32.38	Hex-4-yn-3-one	C6H8O	58.62
**79**	32.39	4-Bromo-2-methoxybut-2-enoic acid, methyl ester	C6H9BrO3	57.18
**80**	32.41	Cyclo(glycyl-L-tryptophan), Ac derivative	C15H15N3O3	57.16
**81**	32.41	9-Octadecenoic acid (Z)-, oxiranylmethyl ester	C21H38O3	58.13
**82**	32.47	10,12-Octadecadienoic acid, 9-oxo-	C18H30O3	63.95
**83**	32.69	1H-Indene, 1-hexadecyl-2,3-dihydro-	C25H42	58.09
**84**	32.80	9,12-Octadecadien-1-ol, (Z,Z)-	C18H34O	83.60

Supplementary Table S2 GC-MS analysis of *Konjac* petroleum ether extract. The table shows the major of chemical compounds isolated from the *Konjac* petroleum ether extract. (Chunyu Wu et al., 2018)

No	RT (min)	Name	Molecular Formula	Match Factor
**85**	33.28	Pentadecanal-	C15H30O	92.20
**86**	33.42	cis-13,16-Docasadienoic acid	C22H40O2	94.75
**87**	33.96	cis-13,16-Docasadienoic acid, methyl ester	C23H42O2	86.89
**88**	34.33	Nitrous oxide	N2O	63.28
**89**	34.76	11,14-Eicosadienoic acid	C20H36O2	79.90
**90**	35.87	1-Cyclohexyldimethylsilyloxybutane	C12H26OSi	62.52

Supplementary Table S2 GC-MS analysis of *Konjac* petroleum ether extract. The table shows the major of chemical compounds isolated from the *Konjac* petroleum ether extract.

No.	Structure	Name	Molecular Formula
1	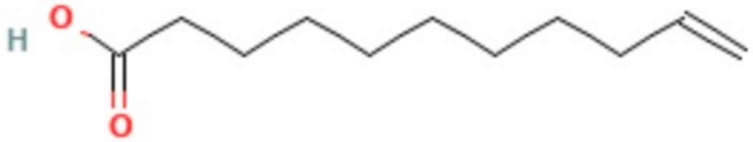	Undecenoic acid	$C_{11}H_{20}O_2$
2	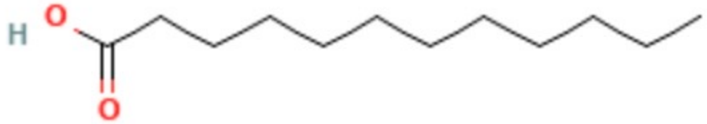	Dodecanoic acid	$C_{12}H_{24}O_2$
3	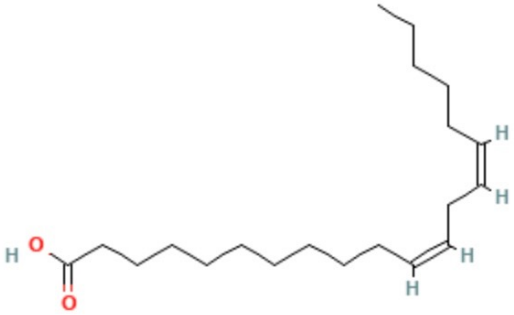	Eicosadienoic acid	$C_{20}H_{36}O_2$
4	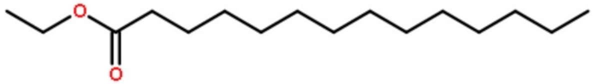	Tetradecanoic acid, ethyl ester	$C_{16}H_{32}O_2$
5	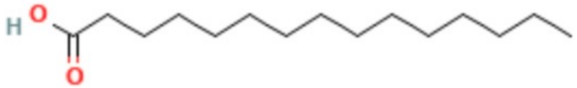	Pentadecanoic acid	$C_{15}H_{30}O_2$

No.	Structure	Name	Molecular Formula
6	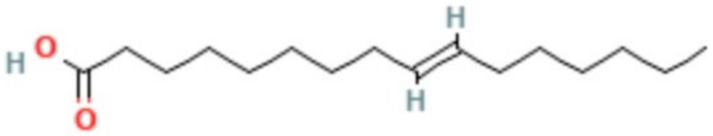	(E)-Hexadec-9-enoic acid	$C_{16}H_{30}O_2$
7	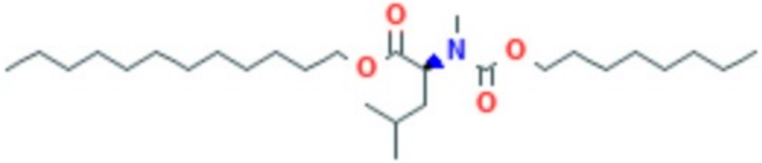	L-Leucine, N-methyl-N-(octyloxycarbonyl)-, dodecyl ester	$C_{28}H_{55}NO_4$
8	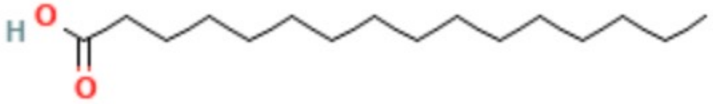	*n*-Hexadecanoic acid	$C_{16}H_{32}O_2$
9	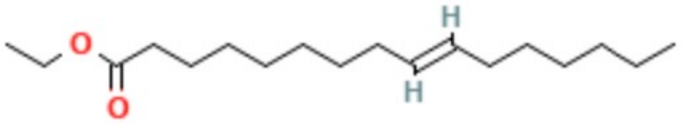	Ethyl 9-hexadecenoate	$C_{18}H_{34}O_2$
10	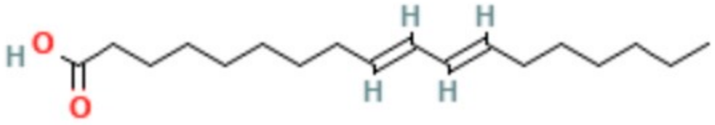	(9E,11E)-Octadecadienoic acid	$C_{18}H_{32}O_2$

*Images of the chemical structures of the bioactive compounds in *Konjac* ether extract were obtained from the PubChem database and Chemical Book database.

Supplementary Figure S3 GC-MS analysis of *Konjac* petroleum ether extract. This figure presents the TIC curve, which represents the cumulative sum of all ion intensities over time, or the number of scans conducted within a specified mass range.

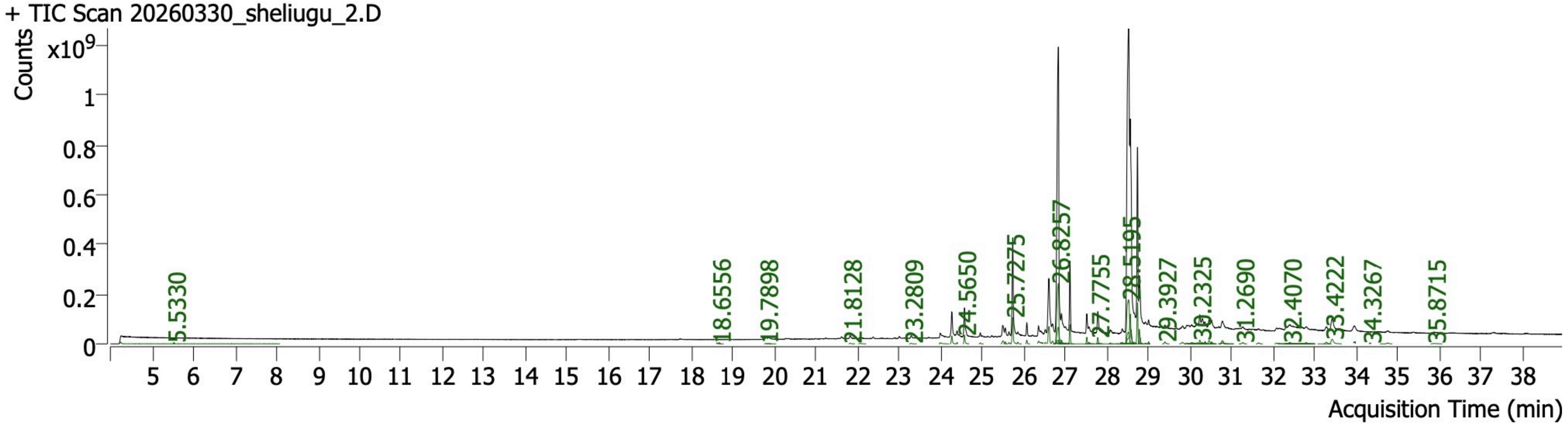

The petroleum ether extract of *Konjac* was analyzed using an Agilent 7890A gas chromatograph coupled with an Agilent 5975 mass selective detector, equipped with an HP-5 capillary column (60 m × 0.32 mm × 0.25 μm; Agilent Technologies). Briefly, 1 μL of the extract solution was injected into a split/splitless inlet at 260 °C. Helium was used as the carrier gas at a flow rate of 1.2 mL/min with a split ratio of 50:1. The column oven temperature was programmed as follows: the initial temperature was set at 50 °C and held for 1 min, then increased to 140 °C at a rate of 5 °C/min, followed by an increase to 240 °C at 10 °C/min, and finally held at 240 °C for 10 min. Analytes were detected in full-scan mode. The mass spectrometry parameters were as follows: electron ionization (EI) mode was employed, with a scan range of m/z 30–400. The electron energy was set to 70 eV, and the ion source temperature was maintained at 260 °C.

Supplementary Figure S4 TLC analysis of *Konjac* petroleum ether extract. The figure shows the major of chemical compounds isolated from the *Konjac* petroleum ether extract.

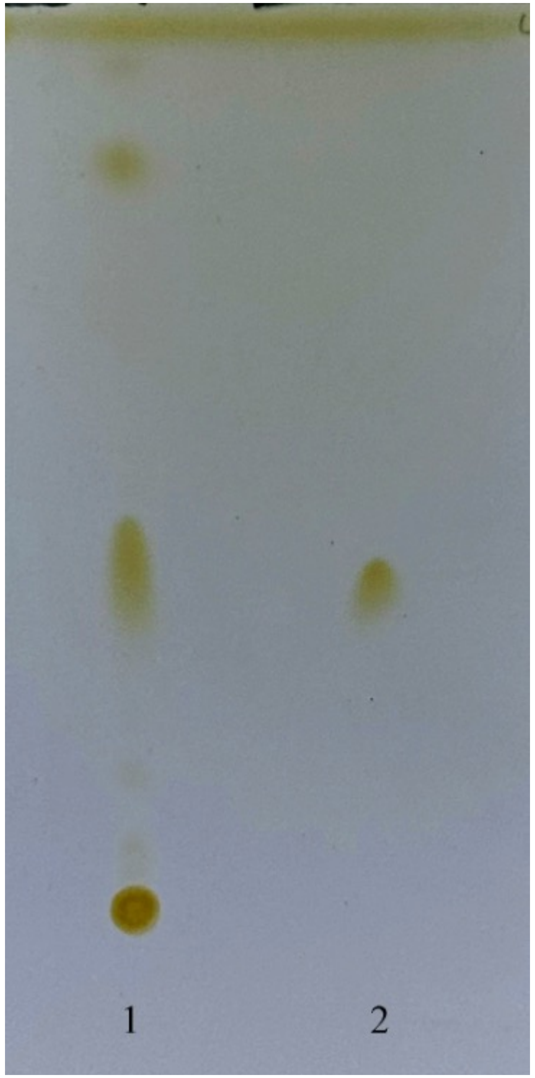

Thin-layer chromatography was performed to qualitatively verify the presence of oleic acid in the petroleum ether extract of Konjac. The qualitative analysis was conducted on HSG-type silica gel plates (Batch No. 20250903, Yantai Jiangyou Silica Gel Development Co., Ltd., Yantai, China) . The test sample was prepared by dissolving the petroleum ether extract of Konjac in petroleum ether at a concentration of 5 mg/mL, while the oleic acid standard (purchased from Sigma-Aldrich, St. Louis, MO, USA) was dissolved in petroleum ether at 2 mg/mL. Both the sample and standard were spotted onto the TLC plate in a volume of 8 μL using a micro-syringe.

Separation was achieved using a mobile phase system consisting of petroleum ether–ethyl acetate–formic acid (95:4:1, v/v). Following development, the chromatogram was visualized by staining with iodine–potassium iodide solution. As illustrated in the TLC profile (Figure S3), the sample spot (Lane 1) co-eluted with the oleic acid standard (Lane 2) at a consistent retention factor (R_f) value. This direct co-chromatographic comparison confirmed the presence of oleic acid in the petroleum ether extract of Konjac.

Notably, the intensity and clarity of the corresponding spot in the sample extract were comparable to that of the oleic acid standard, visually indicating a relatively high content of oleic acid in the extract. This finding was further substantiated by subsequent gas chromatography–mass spectrometry (GC/MS) analysis, which quantified the relative proportion of oleic acid in the total lipid profile of the extract. The combined TLC and GC/MS results thus established oleic acid as a major fatty acid component of the petroleum ether extract from Konjac.

Supplementary Table S4. The quantitative differences in wound healing assay

Name	Area				Healing rate	The remai	Average value	Healing rate	Average healing rate
Y300-OH(1)	40.24		100	78.872		77.91			
						79.167	78.64966667		
			200	81.861		81.233			
						80.689	81.261		
			250	82.46		82.754			
						81.97	82.39466667		
			300	81.116		82.278			
						80.658	81.35066667		
			231	76.076		75.801			
						77.397	76.42466667		
			k0	81.572		81.449			
						80.87	81.297		
		64.328	100	65.328	0.17172127	65.281		0.172317172	
						64.64	65.083	0.180444264	0.174827569
		69.691	200	69.691	0.148666642	68.671		0.161126788	
						70.39	69.584	0.140127778	0.149973736
			250	72.854	0.116492845	71.909		0.127952947	
						69.358	71.37366667	0.158889158	0.134444983
		77.769	300	77.769	0.041261897	78.135		0.03674984	
						77.447	77.78366667	0.04523152	0.041081086
			231	62.328	0.180714023	62.421		0.179491561	
						60.342	61.697	0.206819496	0.18900836
			k0	70.317	0.137976266	71.415		0.124515765	
						70.886	70.87266667	0.131000834	0.131164288
			100	58.27	0.261208033	60.582		0.231894715	
						59.967	59.60633333	0.239692159	0.244264969
		63.709	200	63.709	0.221741733	61.894		0.243913463	
						64.46	63.35433333	0.212567645	0.22607428
		56.891	250	65.891	0.200933786	63.706		0.227431482	
						63.924	64.507	0.224787776	0.217717681
			300	76.349	0.058767691	78.273		0.035048572	
						75.425	76.68233333	0.070158785	0.054658349
			231	49.659	0.34724486	52.116		0.31494821	
						47.036	49.60366667	0.38172354	0.347972203
			k0	68.12	0.164909528	68.836		0.156132006	
						67.483	68.14633333	0.17271858	0.164586705
			100	52.633	0.332678264	51.974		0.341033573	
						52.553	52.38666667	0.333692565	0.335801467
			200	60.273	0.263715322	62.067		0.241800125	
						59.689	60.67633333	0.270849367	0.258788271
			250	64.215	0.221258792	65.65		0.203856415	
						65.832	65.23233333	0.201649285	0.208921497
			300	74.535	0.081130726	72.223		0.109633118	
						74.354	73.704	0.083362099	0.091375314
			231	44.374	0.416714864	40.366		0.469399022	
						41.656	42.132	0.452442295	0.446185394
			k0	63.904	0.216593929	66.422		0.185725494	
						63.697	64.67433333	0.219131565	0.207150329

Supplementary Figure S5. Quantitative statistics of cell invasion

Transwell invasion assay

Cell Counts of Different Visual Fields (Unit: Number)

	231	ko	100	200	300
Up	871	36	1128	147	146
Down	817	82	952	195	75
Left	1029	57	987	223	78
Right	658	48	1076	308	112
Center	942	48	1052	255	101
Average	863	54	1059	225	102

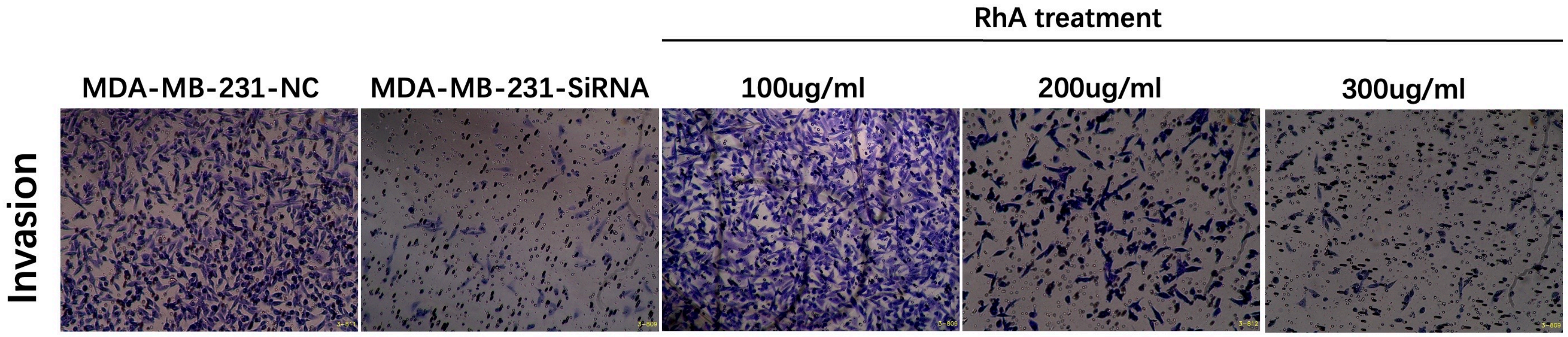

Supplement: Supplementary file 1 [file Supplementaryfile1.pdf]
